# A database of computed Raman spectra of inorganic compounds with accurate hybrid functionals

**DOI:** 10.1038/s41597-024-02924-x

**Published:** 2024-01-22

**Authors:** Yuheng Li, Damien K. J. Lee, Pengfei Cai, Ziyi Zhang, Prashun Gorai, Pieremanuele Canepa

**Affiliations:** 1 Department of Materials Science and Engineering, National University of Singapore, 9 Engineering Drive 1, 117575 Singapore, Singapore; 2 Department of Metallurgical and Materials Engineering, Colorado School of Mines, Golden, Colorado 80401 USA; 3 Department of Chemical and Biomolecular Engineering, National University of Singapore, 4 Engineering Drive 4, 117585 Singapore, Singapore; 4 Department of Electrical and Computer Engineering, University of Houston, Houston, Texas 77204 USA

**Keywords:** Electronic structure, Solid-state chemistry, Density functional theory, Raman spectroscopy

## Abstract

Raman spectroscopy is widely applied in identifying local structures in materials, but the interpretation of Raman spectra is non-trivial. An accurate computational database of reference spectra calculated with a consistent level of theory can significantly aid in interpreting measured Raman spectra. Here, we present a database of Raman spectra of inorganic compounds calculated with accurate hybrid functionals in density functional theory. Raman spectra were obtained by calculating dynamical matrices and polarizability tensors for structures from the Inorganic Crystal Structure Database. The calculated Raman spectra and other phonon properties (e.g., infrared spectra) are stored in a MongoDB database publicly shared through a web application. We assess the accuracy of our Raman calculations by statistically comparing ~80 calculated spectra with an existing experimental Raman database. To date, the database contains 161 compounds and is continuously growing as we add more materials computed with our automated workflow.

## Background & Summary

Raman scattering is the inelastic scattering of light by either absorbing or creating lattice vibrations (phonons)^1^. Raman spectroscopy provides local structure fingerprints of materials by detecting changes in the frequency of incident monochromatic radiation due to the scattering^2,3^. Materials identification with Raman spectroscopy holds advantages including high chemical sensitivity, tolerance to long-range disorder, and high spatial resolution^4,5^. The non-invasive and non-destructive Raman measurement sees applications in *in-situ* and *operando* characterization modes^6^. Compared to other phonon measurement techniques, such as neutron scattering, Raman spectroscopy offers better frequency resolution, easy sample preparation, relatively inexpensive equipment and modularity of lasers, and its fast operation^3–5^. Thanks to these advantages, Raman spectroscopy is widely used in the characterization of biological and organic molecules, as well as inorganic materials in various fields including energy storage and conversion^6–11^, catalysis^12–14^, low-dimensional materials^15,16^, biomedical applications^17–19^, and others. However, the interpretation of experimental Raman spectra may be complicated requiring significant time and effort. This urges the need for accurate computational references to improve the speed and accuracy of Raman spectra’s interpretation.

First-principles calculations of lattice dynamics and phonons have gained considerable attention and are pushing forward the sub-field of computational Raman spectroscopy^20–22^. In 2015, Togo and Tanaka built the well-known first-principles phonon database that contains the full phonon dispersion of a large number of inorganic and ordered materials^23^. This database showcases the power of phonopy -a software package developed to predict the phonon properties of ordered crystals in combination with the accuracy enabled by first-principles calculations^24^. More recently, Liang and co-workers reported a database of computed Raman spectra of approximately 55 compounds derived from density-functional perturbation theory (DFPT) calculations and finite difference derivative of the dielectric tensors^25^. Taghizadeh *et al*. applied time-dependent third-order perturbation theory to calculate Raman spectra of 733 two-dimensional (2D) monolayer materials and used the calculated spectra to identify materials from experimental Raman spectra^26^. Popov and co-workers proposed a new average method for computing Raman spectra of polar polycrystalline materials from DFPT^27^. Most recently, utilizing force constant matrices from the Togo phonon database, Bagheri *et al*. built a Raman database by only calculating Raman tensors by finite differences^28^.

This important progress represents the “bleeding edge” of computational Raman spectroscopy for the purpose of spectra interpretation and further data-driven development. However, one common shortcoming of all these databases is that the predictions are limited to rather low levels of theory, namely the generalized gradient approximation (GGA) exchange-correlation functionals^29,30^, while more accurate hybrid functionals (beyond “plain vanilla” GGA) have not been applied in calculating Raman databases. In particular, the 2D Raman library by Taghizadeh *et al*. is based on the Perdew–Burke–Ernzerhof (PBE) functional^26^, Liang and co-workers’ database was calculated using PBE + *U*^25,31,32^, and the database by Bagheri *et al*. are based on the revised PBE for solids (PBEsol)^28,33^. All these predictions are plagued to a different degree by the pernicious self-interaction error of the semi-local GGA functionals. However, the use of such low-level theories is due to the intrinsic complexity and high computational cost of phonon calculations with higher-level theories, and in particular, their implementation in plane-wave and pseudo-potential codes. Therefore, an efficient and accurate hybrid-functional computational Raman database is in high demand.

In the present data records, we compute Raman spectra together with phonon properties at a consistently high level of theory, namely the hybrid functional PBE0^34^, in a linear combination of atomic orbitals, expanded in terms of triple-ζ valence with polarization (pob-TZVP-rev2) Gaussian basis sets^35,36^. These approximations are realized using the CRYSTAL code at a relatively low computation cost thanks to its exploitation of lattice symmetry during frequency calculations^37–39^. Here, the computation of Raman spectra is automated such that experimentally measured structures are “digested” into the CRYSTAL code, followed by the calculation of Raman spectra and other phonon properties, which are subsequently stored into a MongoDB database. The database is interfaced with an interactive user-friendly web application.

## Methods

### Theory

A small fraction of an incident light beam is scattered when it passes through a substance, or more precisely a material. The inelastic part of the scattering is known as Raman scattering, which changes its frequency by creating or absorbing phonons. From group-theory analysis of crystal symmetry, only particular phonon modes are allowed to take part in Raman scattering. The intensity of the scattering is dependent on the polarizability of materials because the scattering originates from the polarization fluctuations induced by the electric field of the incident electromagnetic radiation. Frequencies of the Raman-active phonon modes and the scattering intensities are calculated to get Raman spectra for inorganic compounds.

Our calculations of Raman spectra are performed using the *ab initio* computational program CRYSTAL^37–39^. The calculations with CRYSTAL have several unique features that facilitate efficient and accurate computation of lattice dynamics: (i) In contrast to most commonly used plane–wave basis sets, the crystalline orbitals are expanded as linear combination of atom centered atomic–orbitals, (ii) the hybrid functional PBE0, with 25% Hartree–Fock exchange^34^, is used rather than the semi-local GGA functionals, such as PBE^30^, and (iii) point group symmetry of lattice is utilized at multiple levels (i.e., atomic positions, wave functions, etc.) during the calculations of vibration frequencies, which means the calculations are only performed on symmetrically irreducible atoms.

**Fig. 1:**
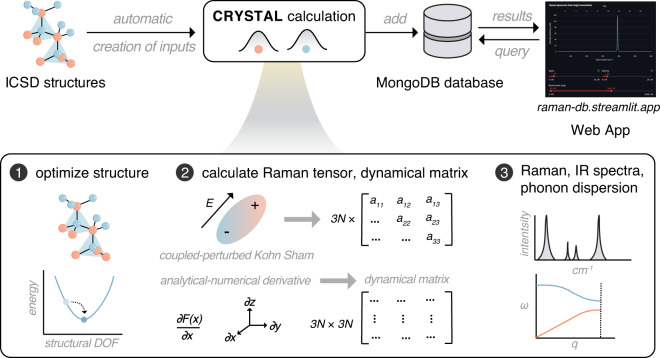
Computational workflow to build the Raman spectra database: structures of inorganic compounds are from the ICSD experimental database; the structures are automatically converted to CRYSTAL input files; CRYSTAL calculations include structure optimization, coupled-perturbed Kohn-Sham for Raman tensors, and numerical differentiation of analytical first derivative of energy versus displacement of each atom to get dynamical matrices; the resulting vibration frequencies and Raman intensities yield Raman spectra; the calculations can also produce phonon dispersion and density of states, as well as Infrared (IR) spectra; after CRYSTAL calculations, the processed outputs are stored in the MongoDB database and interfaced through the web application (https://raman-db.streamlit.app/).

Vibration frequencies are determined in CRYSTAL by calculating and diagonalizing mass-weighted dynamical matrices (bottom box in Fig. 1)^40,41^. The dynamical matrix is a 3*n* × 3*n* (*n* = number of atoms, 3*n* is the number of vibrational normal modes) matrix with components being the second partial derivatives of the DFT total energies versus positional displacements, which are calculated by numerical differentiation of the analytical gradient of the DFT total energy with respect to the atomic positions. Diagonalization of an appropriately symmetrized mass-weighted dynamical matrix then gives the normal modes (eigenvectors) and vibration frequencies (eigenvalues).

In CRYSTAL, Raman intensities are calculated directly through the Raman polarizability tensors obtained via perturbation theory, i.e. the coupled-perturbed Kohn-Sham (CPKS) approach (middle box in Fig. 1)^42–46^. In this approach, a static electric field is applied along different directions to calculate the first-, the second-, and the third-order electric susceptibility tensors, which can be converted into polarizability and dielectric tensors, the first hyperpolarizability tensor, and second hyperpolarizability tensor, respectively. From the electric susceptibility tensors, a 3 × 3 Raman tensors are calculated for each of the 3*n* normal modes. The Raman intensities can then be calculated using the following equations:

1 $${I}_{i,j}(n)=VX{(n,i,j)}^{2},$$

where *I*_*ij*_(*n*) is the directional Raman intensity for single crystals, *X*(*n*, *i*, *j*) is the *i*, *j*-th component of the Raman tensor expressed in the basis of normal mode *n*, and *V* is cell volume. Placzek rotation invariants^5^ are used for averaging the mode Raman tensors of single crystals with Eqs. 2–4:

2 $${G}_{0}(n)=\frac{1}{3}{\left[\sum _{i}X(n,i,i)\right]}^{2},$$

3 $${G}_{1}(n)=\frac{1}{2}\sum _{i,j}{\left[X(n,i,j)+X(n,j,i)\right]}^{2},$$

4 $${G}_{2}(n)=\frac{1}{3}\sum _{i,j}{\left[X(n,i,i)-X(n,j,j)\right]}^{2}.$$

The parallel, perpendicular, and total Raman intensities of powders of polycrystalline materials are computed using Eqs. 5–7:

5 $${I}_{{\rm{par}}}(n)=V\left[15{G}_{0}(n)+6({G}_{1}(n)+{G}_{2}(n))\right],$$

6 $${I}_{{\rm{perp}}}(n)=\frac{9}{2}V\left[{G}_{1}(n)+{G}_{2}(n)\right],$$

7 $${I}_{{\rm{t}}{\rm{o}}{\rm{t}}}(n)={I}_{{\rm{p}}{\rm{a}}{\rm{r}}}(n)+{I}_{{\rm{p}}{\rm{e}}{\rm{r}}{\rm{p}}}(n).$$

Note that intensities can be conveniently recalculated from available Raman tensors with different measurement conditions including temperature and the incident laser frequency of the Raman instrument. Intensities for infrared (IR) absorption are also calculated in the CPKS process and conveniently stored in our database. Symmetry and group theory are applied in the calculations to differentiate Raman- and IR-active vibrational modes.

### Workflow

The development of the present database consisted of three major phases: I. Collecting crystal structures and automatically generating input files for CRYSTAL, II. performing first-principles CRYSTAL calculations, troubleshooting their successful completion followed by data collection, and III. curation of the data with a MongoDB database interfaced by a user-friendly website (Fig. 1).

In Phase I, we first select high-quality experimental structures characterized at low temperature (or room temperature) and ambient pressure from the Inorganic Crystal Structure Database (ICSD)^47^. Then, the experimental structures (i.e., lattice constants, atomic positions, and space groups) are automatically converted into CRYSTAL inputs by symmetry analysis. Specifically, space group and irreducible-atom information needed for CRYSTAL is extracted using the spglib interfaced with the pymatgen library^48,49^. Exceptions are the monoclinic and orthorhombic structures, which may present alternative definitions of unique axes or origins, and they are processed separately. Rhombohedral structures from ICSD usually take their hexagonal conventional cells, and they are all converted to standard rhombohedral primitive cells for consistent input^50^.

In Phase II of our workflow, CRYSTAL calculations are performed for Raman and IR spectra and other phonon-related properties (e.g., phonon dispersion and density of states, etc.). The calculation includes three parts, namely a full structure optimization (i.e., atomic position, lattice parameter, and volume), calculation of vibration frequencies (IR and Raman), and CPKS calculation of Raman intensities. A quasi-Newton algorithm is used for the optimization of atomic positions, cell parameters, and volumes. Consistent and high-accuracy settings are used for all calculations. The DFT total energy was converged to 10^−11^ Hartree/cell (~2.7 × 10^−10^ eV/cell) for the self-consistent field (SCF) procedure. The DFT total energy was integrated with the Pack-Monkhorst sampling scheme over large and symmetrized 8 × 8 × 8 *k*-points grids (SHRINKING = 8). Tolerances for Coulomb and exchange integral series were set to 10^−7^ Hartree for both Coulomb overlap and penetration, 10^−7^ Hartree for exchange overlap, and 10^−9^ and 10^−30^ Hartree for exchange penetration. Crystalline orbitals were expanded as a linear combination of atomic orbitals, which are described by Gaussian valence triple-*ζ* with polarization (pob-TZVP-rev2) basis sets^35,36^. In DFT the unknown exchange and correlation functional was approximated with the PBE0 hybrid functional, which provides excellent predictions of the experimental Raman spectra of Na_3_PS_4_ and quartz-SiO_2_ (Tables 1, 2)^34^.

**Table 1:** Computational Raman frequencies (in cm^−1^) for Na_3_PS_4_ ($P\overline{4}{2}_{1}c$, ICSD No. 121566) calculated with different levels of theory compared to experimental Raman frequencies measured at 100 K.

Mode No.	Mode Symmetry	PBE0	HSE06	R^2^SCAN	PBE0-D3	PBEsol	PBE	Exp.^10^
4–5	E	25	25	31	33	28	27	32
6	A1	71	71	79	77	70	67	70
7–8	E	72	72	80	92	80	71	84
9	B2	87	86	100	111	97	84	84
10–11	E	97	97	102	114	99	94	—
12	B1	100	100	105	116	100	96	—
13–14	E	120	120	125	127	117	116	—
15–16	E	123	123	131	154	137	120	—
18	A1	140	139	146	165	149	135	—
19–20	E	152	152	158	171	158	148	—
21–22	E	156	156	161	173	161	151	157
23	B1	157	156	162	188	164	152	157
24	B2	169	170	175	198	177	165	—
26	B1	175	175	180	207	184	170	—
27–28	E	196	196	200	218	198	188	—
29–30	E	198	197	203	224	203	190	—
31	A1	210	210	211	227	208	202	214
33	B1	227	226	227	239	219	216	226
34	B2	229	229	229	242	221	218	226
35	B1	272	271	269	280	261	258	276
36	B2	272	271	271	283	261	258	276
37–38	E	283	282	282	293	272	268	283
39–40	E	284	283	283	296	273	270	293
42	A1	415	413	404	421	392	387	413
43	B2	538	535	517	546	507	497	537
44–45	E	539	536	517	547	507	498	537
46	B1	569	567	552	579	541	531	567
47–48	E	571	568	553	580	542	533	570
Mean Absolute Error		3 [2]	3 [2]	8 [9]	11 [12]	14 [16]	17 [21]	—
Maximum Absolute Error		12 [9]	12 [10]	20 [20]	31 [31]	30 [30]	40 [40]	—
Mean Absolute Percentage Error		3% [1%]	3% [1%]	4% [2%]	7% [5%]	5% [4%]	6% [6%]	—

Mode number and mode symmetry are from calculations on the PBE0 level. The statistics in square brackets are for the frequencies >100 cm^−1^.

**Table 2:** Computational Raman frequencies (in cm^−1^) for *α*-quartz SiO_2_ (*P*3_2_21, ICSD No. 156197) calculated with different levels of theory and compared to experimental Raman frequencies.

Mode No.	Mode Symmetry	PBE0	HSE06	R^2^SCAN	PBE0-D3	PBEsol	PBE	Exp.^56^
4–5	E	127	126	134	135	125	120	128
6	A1	212	211	240	247	228	206	206
7–8	E	254	254	261	264	246	242	263
9	A1	334	334	329	332	309	314	354
11–12	E	372	372	370	373	351	353	394
13–14	E	428	427	432	440	406	400	402
15	A1	450	450	460	469	440	428	464
17–18	E	686	685	689	702	669	654	697
20–21	E	787	786	789	803	770	753	797
—	(E)	—	—	—	—	—	—	809*
22–23	E	1072	1071	1054	1071	1023	1010	1066*
25	A1	1090	1089	1074	1092	1044	1031	1083
26–27	E	1157	1156	1133	1150	1100	1096	1161
—	(E)	—	—	—	—	—	—	1232*
Mean Absolute Error		11	12	16	14	30	34	—
Maximum Absolute Error		26	26	35	41	61	64	—
Mean Absolute Percentage Error		3%	3%	4%	4%	6%	6%	—

Mode number and mode symmetry are from calculations at the PBE0 level. The experimental frequencies marked with * show barely identifiable intensities as reported in the original spectrum^56^. Their mode symmetries assigned in the experiment are indicated in parentheses when our calculations do not show the mode. Furthermore, the vibration at ~809 cm^−1^ belongs to the degenerate symmetry mode E and can be matched with modes 20–21 indexed by the same symmetry.

At the Γ-point, 3*n* + 1 (with *n* = number of atoms) total energy and gradient calculations are required for computing the IR and Raman frequencies. The first derivatives of the DFT total energies versus atomic displacements, i.e., the total energy gradients to displacements, are computed with a single ionic displacement (0.003 Å) for each coordinate with respect to their equilibrium position. Born charges are also obtained during the frequency calculations. Based on the input generation in Phase I, all calculations are performed in an automatic and high-throughput manner.

In Phase III, this workflow deals with the post-processing of CRYSTAL outputs and subsequent data curation. Output files are parsed to extract relevant data, such as IR and Raman frequencies, and their intensities. The post-processing also includes a convolution routine to generate Raman spectra based on the Voigt model with adjustable percentages of the Gaussian and the Lorentzian shapes. The computed data can be found in Table 3. All data is stored and organized using a MongoDB database currently hosted in the MongoDB Atlas cloud service. An user-friendly and publicly available web app is built for users to search for compounds and plot their Raman spectra interactively (https://raman-db.streamlit.app/).

**Table 3:** Description of the name key, data type, and size for the computational properties as stored in the present database using a nested JSON structure.

Key	Datatype	Size	Description
natoms	scalar	—	Number of atoms
energetics	array	5	Total DFT energy, zero-point energy, *pV*, *TS*, and heat capacity
dielectric	array	3 × 3	Dielectric tensor (First-order electric susceptibility or polarizability)
electric_sus_2	array	3 × 3 × 3	Second-order electric susceptibility tensor (or first hyperpolarizability)
electric_sus_3	array	3 × 3 × 3 × 3	Third-order electric susceptibility tensor (or second hyperpolarizability)
tens_ir	array	3natoms × 3	Infrared (IR) tensor
tens_raman	array	3natoms × 6	Raman tensor (six independent tensor components for each mode)
intens_raman_single	array	3natoms × 3 × 3	Raman intensities for single crystal, which is a 3 × 3 tensor for each mode
intens_raman_multi	array	1D vector	Raman intensities for polycrystal
born	array	natoms × 3 × 3	Born charge matrix for each atom
born_trace	array	natoms	Born charge trace for each atom
born_mode	array	3natoms × 3	Born charge in the basis of normal modes
dyn_matrix	array	3natoms × 3natoms	Symmetric dynamical matrix
vib_freq	array	3natoms	Vibration frequencies (eigenvalues of dyn_matrix)
mode_symmetry	string	—	Symmetry of vibrational modes (irreducible representation)
spectra_raman	array	1D vector	Raman spectra processed via a Voigt convolution
incident_laser	scalar	—	Incident laser wavelength (in nm) used in the calculation
measure_temp	scalar	—	Measurement temperature (in K) used in the calculation
q_points	dict	—	Q-points and coordinates in reciprocal lattice
phonon_dispersion	array	vector	Frequency versus wave vector
phonon_tdos	array	1D vector	Total phonon density of states versus energy/frequency
phonon_pdos	array	1D vector	Projected phonon density of states versus energy/frequency
spectra_ins	array	1D vector	Inelastic neutron scattering spectra, intensity versus energy transfer

## Data Records

The computational data is organized in JSON format and stored in a MongoDB database, which is available in our public GitHub repository (https://github.com/caneparesearch/project Raman) and on Zenodo^51^. The computed IR and Raman frequencies, Raman intensities, and other phonon-related properties for any calculated compounds can be accessed directly from the repository. The names and quantities for the computed properties are stored as key-value pairs using a nested JSON structure, and they are elaborated in Table 3. For each computed compound, the database includes the structure from ICSD and the DFT-optimized structures, the DFT total energies, the vibrational entropy, the heat capacity, the electric susceptibilities up to the third order, the Raman and the IR tensors, the Raman intensities, the Born charges, the dynamical matrices, the vibration frequencies, and their modes’ symmetries. The convoluted Raman and IR spectra obtained from the computations, as well as the simulated measurement temperature and incident laser wavelength, are also included. While the current database includes only Γ-point phonons, the database is designed to be compatible with calculations at different *Q* points other than the Γ-point, phonon dispersions, phonon density of states (DOS), and inelastic neutron scattering (INS) spectra.

**Fig. 2:**
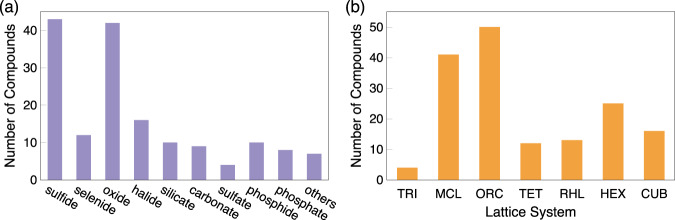
Database statistics. (**a**) Number of compounds for different chemistries; the selenide bar also includes telluride-based compounds, and the phosphate bar also includes thiophosphate-based compounds; “others” include nitride, nitrate, borate, and carbide. (**b**) Number of compounds for the seven lattice systems: triclinic (TRI), monoclinic (MCL), orthorhombic (ORC), tetragonal (TET), rhombohedral (RHL), hexagonal (HEX), and cubic (CUB).

The database currently contains 161 calculated inorganic compounds. Figure 2 shows the statistics of the database: There are 43 sulfides, 12 selenides and telluriums, 42 oxides, and peroxides, 16 halides, 10 silicates, 9 carbonates, 4 sulfates, 10 phosphides, 8 phosphates, and thiophosphates, and the remailing 7 compounds are nitrides, nitrates, borates, and carbides, respectively. Most compounds belong to the monoclinic (41) and orthorhombic (50) lattice systems, and there are also 4 triclinic, 12 tetragonal, 13 rhombohedral, 25 hexagonal, and 16 cubic systems, respectively. The 161 compounds computed are listed in Tables 4, 5 with their ICSD codes, space group symbols, and a link to their detailed Raman information (computed outputs), including the calculated Raman- (and IR-) active vibrational modes and intensities.

**Table 4:** List of computed compounds (No. 1–90) in the database and their Raman information, such as Raman-active frequencies and Raman intensities.

Compound [ICSD, S.G.]	Raman Table	Compound [ICSD, S.G.]	Raman Table
Al_2_SiO_5_ [84613, 58]	Al2SiO5_58_icsd84613	P_2_S_3_ [423037, 11]	P2S3_11_icsd423037
BaTi(SiO_3_)_3_ [18100, 188]	BaTi(SiO3)3_188_icsd18100	Ca_2_ZnSi_2_O_7_ [186944, 113]	Ca2ZnSi2O7_113_icsd186944
Sb_2_O_3_ [240206, 227]	Sb2O3_227_icsd240206	P_4_S_3_ [406048, 62]	P4S3_62_icsd406048
Li_3_PO_4_ [257440, 62]	Li3PO4_62_icsd257440	P_2_S_5_ [409061, 2]	P2S5_2_icsd409061
Sb_2_O_3_ [414463, 19]	Sb2O3_19_icsd414463	CoS_2_ [86351, 205]	CoS2_205_icsd86351
NaS [73173, 194]	NaS_194_icsd73173	HgS [81923, 152]	HgS_152_icsd81923
Na_2_O_2_ [134060, 189]	Na2O2_189_icsd134060	MgTiO_3_ [55285, 148]	MgTiO3_148_icsd55285
BeAl_2_O_4_ [72416, 62]	BeAl2O4_62_icsd72416	NaP_5_ [99177, 62]	NaP5_62_icsd99177
Mg_3_(BO_3_)_2_ [31385, 58]	Mg3(BO3)2_58_icsd31385	NaP_15_ [239084, 2]	NaP15_2_icsd239084
P_2_O_3_ [24407, 11]	P2O3_11_icsd24407	CoAsS [69129, 29]	CoAsS_29_icsd69129
Na_2_Mg(CO_3_)_2_ [100482, 148]	Na2Mg(CO3)2_148_icsd100482	As_4_S_5_ [16107, 11]	As4S5_11_icsd16107
FeAsS [185809, 14]	FeAsS_14_icsd185809	P2S_7_ [423062, 2]	P2S7_2_icsd423062
P_2_Se_5_ [74546, 14]	P2Se5_14_icsd74546	Si_3_(BiO_3_)_4_ [69430, 220]	Si3(BiO3)4_220_icsd69430
BeO [15620, 186]	BeO_186_icsd15620	ZnO_2_ [38979, 205]	ZnO2_205_icsd38979
Li_4_P_2_O_7_ [130427, 153]	Li4P2O7_153_icsd130427	Na_3_PS_4_ [121567, 114]	Na 3 PS 4 _114_icsd121567
TeO_2_ [26844, 61]	TeO2_61_icsd26844	LiP [100465, 14]	LiP_14_icsd100465
As_2_S_3_ [15239, 14]	As2S3_14_icsd15239	CuSbS_2_ [171051, 62]	CuSbS2_62_icsd171051
PS [1703, 15]	PS_15_icsd1703	BaBe_2_Si_2_O_7_ [100030, 62]	BaBe2Si2O7_62_icsd100030
Sb_2_Te [69557, 164]	Sb2Te_164_icsd69557	P_2_S_7_ [423061, 14]	P2S7_14_icsd423061
Te_3_As_2_ [18208, 12]	Te3As2_12_icsd18208	AsSe [2056, 14]	AsSe_14_icsd2056
FeSbS [24161, 14]	FeSbS_14_icsd24161	TiO_2_ [9161, 136]	TiO2_136_icsd9161
AsS [86228, 15]	AsS_15_icsd86228	Li_3_P [240861, 194]	Li3P_194_icsd240861
ZnS [67453, 186]	ZnS_186_icsd67453	ZrO_2_ [86692, 14]	ZrO2_14_icsd86692
As_2_O_3_ [238641, 14]	As2O3_14_icsd238641	Li_5_Ge_2_ [121533, 166]	Li5Ge2_166_icsd121533
TiO_2_ [9852, 141]	TiO2_141_icsd9852	HgO [14124, 62]	HgO_62_icsd14124
FeS_2_ [42415, 58]	FeS2_58_icsd42415	Li_4_GeS_4_ [95649, 62]	Li4GeS4_62_icsd95649
As_2_S_3_ [185819, 2]	As2S3_2_icsd185819	PbO [15466, 129]	PbO_129_icsd15466
GeS_2_ [44, 7]	GeS2_7_icsd44	Zn(AsO_2_)_2_ [202249, 14]	Zn(AsO2)2_14_icsd202249
LiI [414242, 186]	LiI_186_icsd414242	PbSeO_3_ [98376, 11]	PbSeO3_11_icsd98376
Na_3_PO_4_ [97205, 114]	Na3PO4_114_icsd97205	SbCl_3_ [8258, 62]	SbCl3_62_icsd8258
CdS [154186, 186]	CdS_186_icsd154186	ZnSe [77091, 216]	ZnSe_216_icsd77091
ZnO [44477, 186]	ZnO_186_icsd44477	As_4_S_3_ [16105, 62]	As4S3_62_icsd16105
CuAgS [66580, 36]	CuAgS_36_icsd66580	Na_3_PS_4_ [121566, 114]	Na 3 PS 4 _114_icsd121566
NaS_2_ [2586, 122]	NaS2_122_icsd2586	As_8_S_9_ [98792, 13]	As8S9_13_icsd98792
Al_2_ZnO_4_ [185709, 227]	Al2ZnO4_227_icsd185709	HgS [38471, 154]	HgS_154_icsd38471
AsS_2_ [424590, 4]	AsS2_4_icsd424590	Ba(NO_3_)_2_ [35495, 205]	Ba(NO3)2_205_icsd35495
PbCO_3_ [166089, 62]	PbCO3_62_icsd166089	ZnTe [77072, 216]	ZnTe_216_icsd77072
Sb_2_O_3_ [2033, 56]	Sb2O3_56_icsd2033	P4S_5_ [1995, 11]	P4S5_11_icsd1995
PO_2_ [42777, 15]	PO2_15_icsd42777	Li_3_PO_4_ [77095, 62]	Li3PO4_62_icsd77095
SbO_2_ [153156, 15]	SbO2_15_icsd153156	BaCO_3_ [158378, 62]	BaCO3_62_icsd158378
CaTa_2_O6 [24091, 62]	CaTa2O6_62_icsd24091	Li_2_O [257372, 225]	Li2O_225_icsd257372
Ca_3_Si_2_O7 [2282, 14]	Ca3Si2O7_14_icsd2282	Sb_2_Se_3_ [30973, 62]	Sb2Se3_62_icsd30973
Al_2_Si(O_2_F)_2_ [59410, 62]	Al2Si(O2F)2_62_icsd59410	TiO_2_ [36408, 61]	TiO2_61_icsd36408
ZnS [136503, 216]	ZnS_216_icsd136503	Ag_2_HgS_2_ [201713, 14]	Ag2HgS2_14_icsd201713
PbClF [39165, 129]	PbClF_129_icsd39165	GeP_3_ [16294, 166]	GeP3_166_icsd16294

The database currently contains 161 compounds and constantly grows. ICSD codes and space group (S.G.) numbers, of compounds are provided.

**Table 5:** List of computed compounds (No. 91–161) in the database and their Raman information, such as Raman-active frequencies and Raman intensities.

Compound [ICSD, S.G.]	Raman Table	Compound [ICSD, S.G.]	Raman Table
ZnF_2_ [280605, 136]	ZnF2_136_icsd280605	AsS [360, 14]	AsS_14_icsd360
SiO_2_ [90145, 152]	SiO2_152_icsd90145	LiP_5_ [88710, 33]	LiP5_33_icsd88710
Li_3_P_7_ [60774, 19]	Li3P7_19_icsd60774	CaMgCO_32_ [52149, 148]	CaMgCO32_148_icsd52149
Bi_2_Se_3_ [60205, 62]	Bi2Se3_62_icsd60205	K_3_Na(SO_4_)_2_ [26014, 164]	K3Na(SO4)2_164_icsd26014
ZnP_4_ [40428, 92]	ZnP4_92_icsd40428	KNbO_3_ [190923, 160]	KNbO3_160_icsd190923
NaHCO_3_ [18183, 14]	NaHCO3_14_icsd18183	NaMgF_3_ [72318, 62]	NaMgF3_62_icsd72318
ZnAs [427612, 61]	ZnAs_61_icsd427612	Mg_3_(PO_4_)_2_ [31005, 14]	Mg3(PO4)2_14_icsd31005
As_2_Se_3_ [29535, 14]	As2Se3_14_icsd29535	SiC [156190, 186]	SiC_186_icsd156190
CaTiO_3_ [62149, 62]	CaTiO3_62_icsd62149	Ag_3_AsS_3_ [36352, 15]	Ag3AsS3_15_icsd36352
CaCO_3_ [15194, 62]	CaCO3_62_icsd15194	As_2_O_3_ [238665, 14]	As2O3_14_icsd238665
Li_3_N [156894, 191]	Li3N_191_icsd156894	BaSO_4_ [33730, 62]	BaSO4_62_icsd33730
NaP [14009, 19]	NaP_19_icsd14009	Li_2_O_2_ [50658, 194]	Li2O2_194_icsd50658
SbI_3_ [30906, 14]	SbI3_14_icsd30906	CoAsS [610107, 198]	CoAsS_198_icsd610107
AsO_2_ [10436, 62]	AsO2_62_icsd10436	FeS_2_ [316, 205]	FeS2_205_icsd316
CuBiS_2_ [34936, 62]	CuBiS2_62_icsd34936	Sb_2_O_5_ [1422, 15]	Sb2O5_15_icsd1422
P2O_5_ [79698, 62]	P2O5_62_icsd79698	CaMg_3_(CO_3_)_4_ [201729, 155]	CaMg3(CO3)4_155_icsd201729
AsBr_3_ [26774, 19]	AsBr3_19_icsd26774	LiCaAlF_6_ [150332, 163]	LiCaAlF6_163_icsd150332
Sb_2_PbO_6_ [81387, 162]	Sb2PbO6_162_icsd81387	As_2_Se_3_ [291473, 12]	As2Se3_12_icsd291473
Ca_2_CO_3_F_2_ [100607, 60]	Ca2CO3F2_60_icsd100607	SiO_2_ [403365, 92]	SiO2_92_icsd403365
PbSO_4_ [16916, 62]	PbSO4_62_icsd16916	NaBe_4_SbO_7_ [27599, 186]	NaBe4SbO7_186_icsd27599
KAlSiO_4_ [83449, 159]	KAlSiO4_159_icsd83449	RuS_2_ [68472, 205]	RuS2_205_icsd68472
BaBe_2_Si_2_O_7_ [20409, 31]	BaBe2Si2O7_31_icsd20409	BaSi_2_O_5_ [100313, 62]	BaSi2O5_62_icsd100313
Ag_2_S [262636, 14]	Ag2S_14_icsd262636	Na_2_Si_2_O_5_ [34688, 14]	Na2Si2O5_14_icsd34688
SbO_2_ [153154, 33]	SbO2_33_icsd153154	PSe [74878, 14]	PSe_14_icsd74878
TlAsS_2_ [81094, 14]	TlAsS2_14_icsd81094	CaCO_3_ [18166, 167]	CaCO3_167_icsd18166
Al_2_SiO_5_ [24275, 58]	Al2SiO5_58_icsd24275	ZnN_6_ [430428, 14]	ZnN6_14_icsd430428
SbCl_5_ [250363, 194]	SbCl5_194_icsd250363	AsI_3_ [23003, 148]	AsI3_148_icsd23003
As_2_O_3_ [238612, 227]	As2O3_227_icsd238612	ZnCl_2_ [2459, 33]	ZnCl2_33_icsd2459
Li_2_S [657596, 225]	Li2S_225_icsd657596	P_4_O_9_ [300205, 167]	P4O9_167_icsd300205
KBF_4_ [9875, 62]	KBF4_62_icsd9875	MgCO_3_ [80870, 167]	MgCO3_167_icsd80870
SbI_3_ [26082, 148]	SbI3_148_icsd26082	Na_2_S_5_ [430333, 32]	Na2S5_32_icsd430333
Na_2_S_5_ [38349, 62]	Na2S5_62_icsd38349	Na_3_P [433838, 185]	Na3P_185_icsd433838
P_4_O_7_ [16452, 14]	P4O7_14_icsd16452	SbBr_3_ [14217, 19]	SbBr3_19_icsd14217
Cu_2_O [172174, 224]	Cu2O_224_icsd172174	Sb_2_Te_3_ [2084, 166]	Sb2Te3_166_icsd2084
WS_2_ [202366, 194]	WS2_194_icsd202366	Na_2_Ca(SO_4_)_2_ [16901, 15]	Na2Ca(SO4)2_15_icsd16901
Li_3_PO_4_ [257439, 31]	Li3PO4_31_icsd257439		

The database currently contains 161 compounds and constantly grows. ICSD codes and space group (S.G.) numbers, of compounds are provided.

## Technical Validation

To validate our simulated Raman spectra obtained with the hybrid functional and local basis sets, we compare these predictions with experimental spectra directly available in the literature or in the RRUFF database -an integrated database of Raman spectra data for minerals^52^.

Computational Raman spectra of two compounds (Na_3_PS_4_ and *α*-quartz SiO_2_) were benchmarked against experimental Raman spectra from the literature to demonstrate the high accuracy of the hybrid functional PBE0 approximation compared to other functionals. Table 1 shows the computed frequencies of Raman-active vibrational modes of Na_3_PS_4_ ($P\overline{4}{2}_{1}c$, ICSD No. 121566) calculated by different approximations of the unknown exchange and correlation functional, and compared with experimental frequencies^10^. The experimentally observed Raman-active modes are matched to computational ones according to their mode symmetries. Errors of predicted frequencies are calculated with respect to the matched experimental frequencies, and they are listed in the last three rows of Table 1. Among all the exchange and correlation functionals tested, the hybrid functional PBE0 used for this dataset shows the smallest mean absolute error (MAE), maximum absolute error (MaxAE), and mean absolute percentage error (MAPE), of only 3 cm^−1^, 12 cm^−1^, and 2.9%, respectively. In contrast, the range–separated hybrid functional by Heyd, Scuseria and Ernzerhof HSE06^53,54^ shows comparable accuracy as PBE0 in determining Raman frequencies, but calculations with HSE06 in CRYSTAL are almost twice more expensive than those with PBE0 (e.g., computation time in seconds is 31,492 vs. 12,059 on 128 cores –2 × AMD EPYC 7742– for Na_3_PS_4_). Notably, PBE0 and HSE06 perform especially well for the prediction of “high”-frequency modes (>100 cm^−1^). The GGA functionals PBE and PBEsol show the worst performance with MAE, MaxAE, and MAPE of up to 17 cm^−1^, 40 cm^−1^, and 6%, respectively. These inaccuracies are not acceptable if computation serves to fingerprint Raman spectra of ordered materials. Meta-GGA functionals in the flavor of R^2^SCAN show relatively small absolute errors compared to GGA, but the inaccuracies compared to experimental data are still approximately twice (2×) that of PBE0. With the van der Waals correction (Grimmes’s D3)^55^, PBE0-D3 shows worse accuracy in frequencies than PBE0 alone, with an 11 cm^−1^ MAE. The worse accuracy when including D3 is partially attributed to the ionic character of the Na^+^–PS_4_^3-^ bonds in Na_3_PS_4_. As shown by the error values in square brackets of Table 1, PBE0 and HSE06 show decreased MAE for the high-frequency modes, while the high-frequency MAE of all the other functionals increases. A decrease in MaxAE from 12 to 9 cm^−1^ from all frequencies to high frequencies for PBE0 also indicates that the max error ≥10 cm^−1^ only appears at low frequencies (<100 cm^−1^). This further encourages Raman calculations using PBE0 because high-frequency vibrational modes appear less noisy than soft modes in experimental measurements, and accurate computational reference of the high-frequency modes are more important in facilitating the interpretation of experimental spectra.

The *α*-quartz SiO_2_ (*P*3_2_21, ICSD No. 156197) is another widely studied compound with an experimental Raman spectrum^56^. Table 2 shows the calculated frequencies with different DFT exchange and correlation functionals and their errors with respect to the experimental reference. Interestingly, three of the reported experimental assignments do not show observable peaks in the Raman spectra from the same study^56^, and two of them are also absent in our calculation results. The three non-observable frequencies are marked with * in the last column of Table 2, and the frequency errors were calculated only for the modes identified in our calculations.

As in the case of Na_3_PS_4_, frequencies calculated with PBE0 appear in good agreement with experimental observations, and thus very accurate. The PBE0 frequencies show the smallest MAE, MaxAE, and MAPE of 11 cm^−1^, 26 cm^−1^, and 3%. Except for the computationally more expensive HSE06, all the other exchange and correlation functionals do not show comparable accuracy to PBE0. Vibrational modes are assigned according to the selection rule as IR-active (change in dipole moment) and Raman-active (change in polarizability) modes.

Due to the lack of high-quality Raman data from consistent measurement in literature, we decided to further validate our computational approach by a systematic comparison of calculated spectra with the experimental Raman database RRUFF^52^. We applied our approach to calculating Raman spectra for 78 inorganic compounds available in the RRUFF database, and these spectra have also been added to our database. To generate input following our workflow, the RRUFF compounds were matched to ICSD structures according to their chemical formula and mineral names. The matching was subsequently verified using space group information of Materials Project^57^ entries that were assigned to RRUFF compounds in Ref. ^28^.

**Fig. 3:**
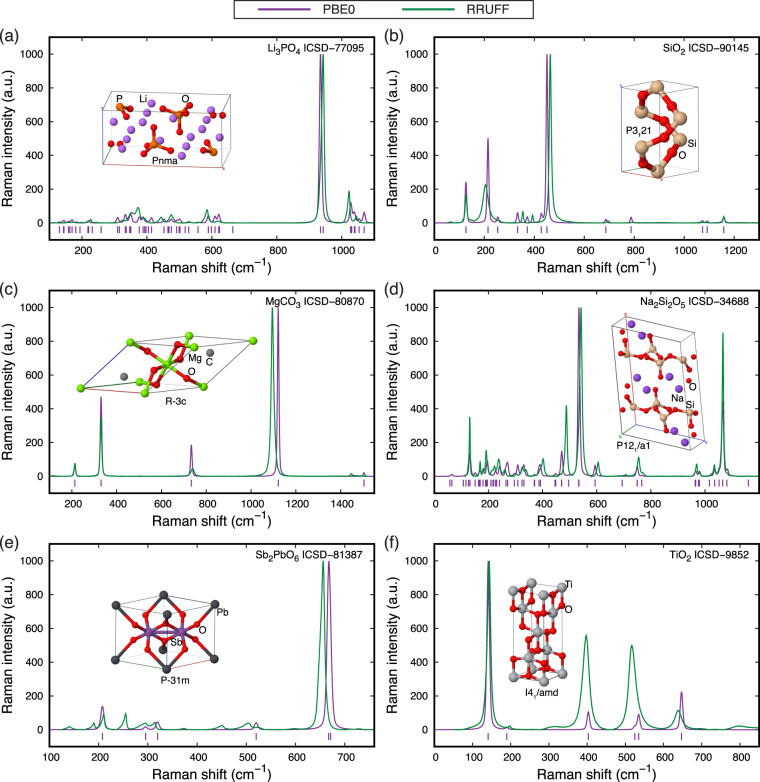
Raman spectra calculated using our workflow with the hybrid functional PBE0 (purple) in comparison with RRUFF experimental spectra (green) for six inorganic compounds: (**a**) Li_3_PO_4_ with identified ICSD No. 77095 and space group *Pnma*, (**b**) SiO_2_ (ICSD No. 90145 and *P*3_1_21), (**c**) MgCO_3_ (ICSD No. 80870 and *R*−3*c*), (**d**) Na_2_Si_2_O_5_ (ICSD No. 34688 and *P*12_1_/*a*1), (**e**) Sb_2_PbO_6_ (ICSD No. 81387 and *P*−31 *m*), and (**f**) TiO_2_ (ICSD No. 9852 and *I*4_1_/*amd*). Raman intensities are in arbitrary units (a.u.) and the maximum peak in each spectrum is normalized to 1000. Raman-active vibration modes are marked as purple ticks at the bottom of each spectrum. All calculated spectra are plotted using a Voigt line shape with 50% Lorentzian and 50% Gaussian. Insets show optimized crystal structures from our calculations. A complete spectra comparison of all the 78 calculated RRUFF compounds can be found in Section S3 of Supporting Information.

Figure 3 shows the comparison of the calculated (purple) and RRUFF experimental (green) Raman spectra for six chosen inorganic compounds of the 78 inorganic compounds considered here: Li_3_PO_4_ (ICSD No. 77095), SiO_2_ (ICSD No. 90145), MgCO_3_ (ICSD No. 80870), Na_2_Si_2_O_5_ (ICSD No. 34688), Sb_2_PbO_6_ (ICSD No. 81387), and TiO_2_ (ICSD No. 9852). The maximum Raman intensities are normalized to 1,000 arbitrary units (a.u.) and plotted versus Raman frequencies in cm^−1^. The calculated spectra are then convoluted with a Voigt line shape consisting of 50% Lorentzian and 50% Gaussian. Purple ticks at the bottom of each spectrum mark the calculated Raman frequencies. Insets show optimized crystal structures from our calculations, which are visualized with JS-ICE^58^ together with their computed space groups. The spectra comparison for all the 78 calculated RRUFF compounds can be found in Supplementary Figures S2–S15 of Supporting Information (SI).

The calculated and experimental Raman spectra of the six compounds in Fig. 3 show very good agreement. As shown in the plots, the experimental peaks are all correctly predicted with very small deviations in the values of frequencies. Specifically, 87 out of the total 93 peaks show wavenumber deviations within ±10 cm^−1^ and 91 peaks within ±15 cm^−1^. The relative deviations are within ±3% for 88 out of the total 93 peaks, and within ±5% for 92 peaks. Predictions are especially accurate for the most intense peak in each spectrum, with a maximum deviation of only ~–9.3 cm^−1^ in the case of MgCO_3_ (Fig. 3c). Note that large deviations in intensities are only observed for relatively experimental weak peaks (Fig. 3f). Such accurate prediction of the most intense peak for a compound is very useful when the prediction is used to fingerprint phases from experimental Raman spectra. Notably, the six spectra (Fig. 3) do not represent the best computation–experiment matches among all the 78 spectra (Supplementary Figures S2–S15 of SI).

Besides the individual spectra comparison for each compound, a statistical comparison was also made between calculated and experimental Raman-active frequencies, to provide a more systematic evaluation of the overall computational accuracy. Among all the 78 RRUFF compounds we calculated, the spectra of 15 compounds show experimental artifacts that make it impossible to compare their frequencies with our calculated Raman-active frequencies (Supplementary Figures S13–S15 of SI), and the spectra of the rest 63 compounds (Supplementary Figures S2–S12 of SI) were used for the statistical comparison. Given a pair of computational and RRUFF experimental spectra for one of the 63 compounds, the common frequency range of the spectra was first identified. Then, within the common range, all local maxima of the Raman intensities and their frequencies were located. Finally, each computational intensity maximum (i.e., a peak in the computational Raman spectrum) is uniquely matched to an experimental intensity maximum (i.e., a peak in the experimental Raman spectrum), and the computational frequency deviation from the experiments is calculated for each pair of the matched Raman peaks.

**Fig. 4:**
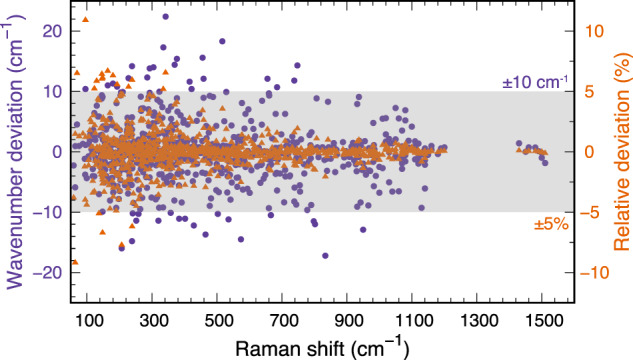
Wavenumber deviation (purple) and relative deviation (orange) of calculated Raman frequencies compared to the RRUFF experimental Raman frequencies. The grey shaded area indicates a wavenumber-deviation range of ±10 cm^−1^ and a relative-deviation range of ±5%. The frequency comparison is only conducted within the common frequency range between computational and experimental spectra. A total number of 63 PBE0-RRUFF spectra are used to extract the deviations (Supplementary Figures S2–S12 of Supporting Information). There are 15 RRUFF spectra found to have experimental artifacts and are not included in this comparison to the computed spectra (Supplementary Figures S13–S15 of Supporting Information).

Figure 4 shows the computational frequency deviations from the matched RRUFF Raman-active frequencies, plotted versus Raman shift. The wavenumber deviation is plotted using purple points, and the relative deviation is plotted using orange triangles. The grey shaded area indicates a wavenumber deviation range of ±10 cm^−1^ and a relative deviation range of ±5%. There are a total number of 804 pairs of matched peaks from the 63 spectra used for comparison. A percentage of 94.7% (88.9%) wavenumber deviations are within the range of ±10 cm^−1^ (±8 cm^−1^), and a percentage of 97.6% (92.9%) relative deviations are within the range of ±5% (±3%). The maximum absolute values of wavenumber and relative deviations are 22.4 cm^−1^ and 10.9%, respectively. More data points are found at smaller Raman shift wavenumbers because the Raman-active modes are overall more frequently distributed at lower frequencies for the 63 compounds (Supplementary Figures S2–S12 of SI). Another trend shown in Fig. 4 is that the deviations are generally larger at lower frequencies. A plausible reason for this trend is that experimental measurements are usually more noisy for softer vibrational modes.

## Usage Notes

**Fig. 5:**
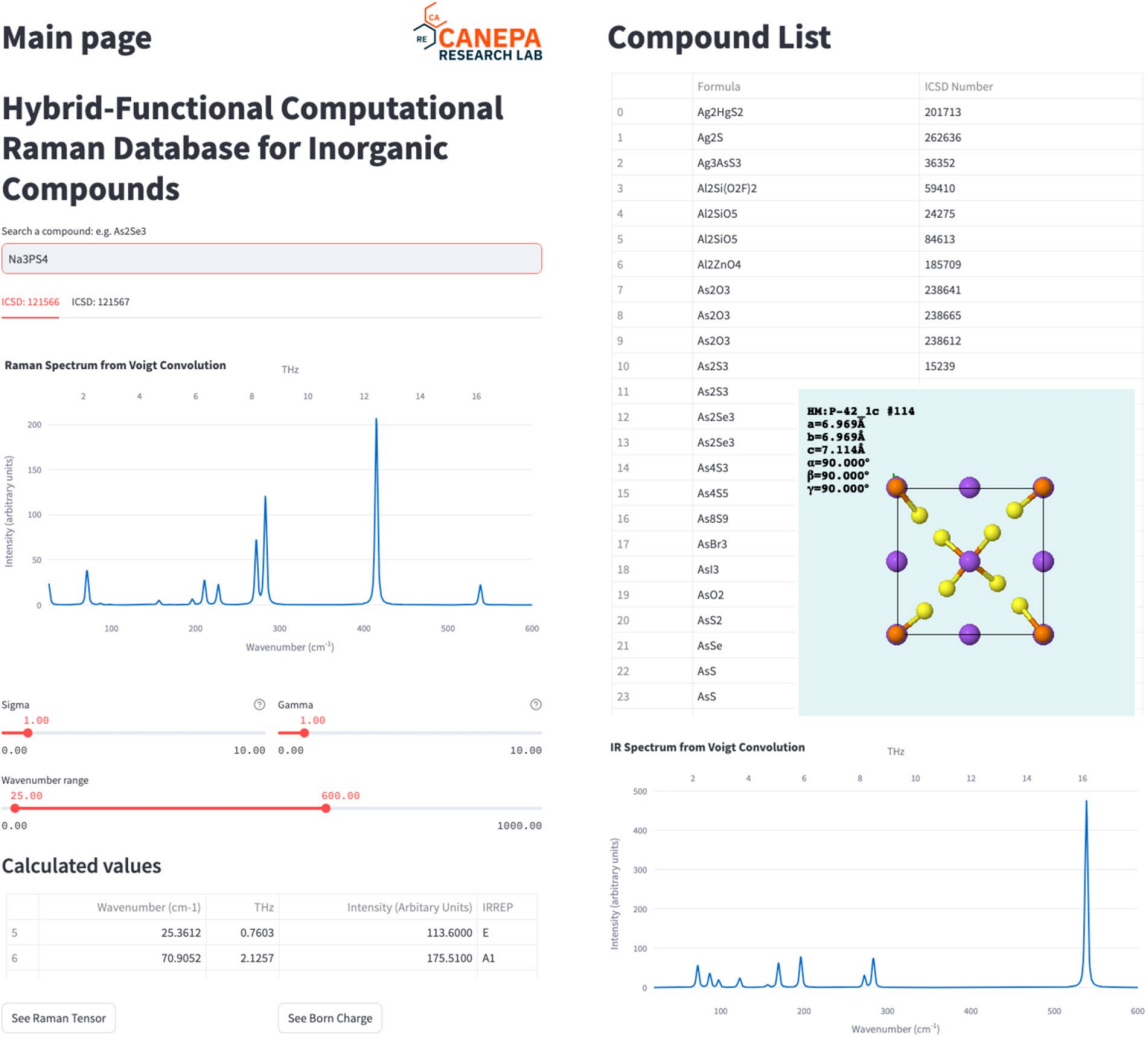
A demo of the Raman-database web application (https://raman-db.streamlit.app/). The database of computed Raman properties is interfaced by this web application in the ways of searching for compounds, viewing crystal structures, interactively plotting Raman and IR spectra, and query for phonon properties (left and bottom right). A complete list of all available inorganic compounds in the database and their ICSD numbers (right). The list currently contains 161 compounds and is growing.

The present database is accessible through our web application (https://raman-db.streamlit.app/). In this web application (Fig. 5), users can search for chemical formulae, select the desired compound according to its ICSD identification number, view the crystal structure, and interactively plot the Raman and IR spectra with different convolution schemes (i.e., Gaussian shapes, Lorentzian shapes, and Voight shapes). The spectra plots can be downloaded as a PNG file, and the spectra data (frequencies and intensities for the Raman- and IR-active vibrational modes, together with their irreducible representations) can be downloaded as practical CSV files. Relevant quantities, such as Raman tensors and Born charges are also available in the application. A complete list of calculated compounds in the present database is constantly updated and shown on the web page.

### Supplementary information

Supplementary Information

## Data Availability

Data and custom code are available on GitHub repository for the automation of Raman predictions, post-processing, and database construction, under a CC BY 4.0 License^51^. The *ab initio* code CRYSTAL is commercially available, and the TZVP basis sets used (see Supplementary Section S1 of the SI) can be accessed from the CRYSTAL website.
