# Supplementary material for: A database of computed Raman spectra of inorganic compounds with accurate hybrid functionals
Source: Sci Data. 2024 Jan 22;11:105. doi: 10.1038/s41597-024-02924-x (PMC10803741; doi:10.1038/s41597-024-02924-x)
Supplement: Supplementary file 1 — Supplementary Information [file 41597_2024_2924_MOESM1_ESM.pdf]

–Supplementary Information– A database of computed Raman spectra of inorganic compounds with accurate hybrid functionals

Yuheng Li[1], Damien K. J. Lee[1], Pengfei Cai[1], Ziyi Zhang[1], Prashun Gorai[2], and Pieremanuele Canepa[1,3,4*]

[1]Department of Materials Science and Engineering, National University of Singapore, 9 Engineering Drive 1, 117575, Singapore
[2]Department of Metallurgical and Materials Engineering, Colorado School of Mines, Golden, Colorado 80401, United States
[3]Department of Chemical and Biomolecular Engineering, National University of Singapore, 4 Engineering Drive 4, 117585, Singapore
[4]Department of Electrical and Computer Engineering, University of Houston, Houston, Texas 77204, United States
[*]corresponding author(s): Pieremanuele Canepa (pcanepa@central.uh.edu)

Contents

Supplementary Figures **2**
Supplementary Tables **17**

List of Figures

1 Comparison of Raman spectra plotted using different line shapes. 2
2 Comparison of calculated (PBE0) and experimental (RRUFF) Raman spectra #1–6 3
3 Comparison of calculated (PBE0) and experimental (RRUFF) Raman spectra #7–12 4
4 Comparison of calculated (PBE0) and experimental (RRUFF) Raman spectra #13–18 5
5 Comparison of calculated (PBE0) and experimental (RRUFF) Raman spectra #19–24 6
6 Comparison of calculated (PBE0) and experimental (RRUFF) Raman spectra #25–30 7
7 Comparison of calculated (PBE0) and experimental (RRUFF) Raman spectra #31–36 8
8 Comparison of calculated (PBE0) and experimental (RRUFF) Raman spectra #37–42 9
9 Comparison of calculated (PBE0) and experimental (RRUFF) Raman spectra #43–48 10
10 Comparison of calculated (PBE0) and experimental (RRUFF) Raman spectra #49–54 11
11 Comparison of calculated (PBE0) and experimental (RRUFF) Raman spectra #55–60 12
12 Comparison of calculated (PBE0) and experimental (RRUFF) Raman spectra #61–63 13
13 Comparison of calculated (PBE0) and experimental (RRUFF) Raman spectra #64–69 14
14 Comparison of calculated (PBE0) and experimental (RRUFF) Raman spectra #70–75 15
15 Comparison of calculated (PBE0) and experimental (RRUFF) Raman spectra #76–78 16

List of Tables

1 List of the TZVP basis sets for elements in the computed inorganic compounds. 17

Supplementary Figures

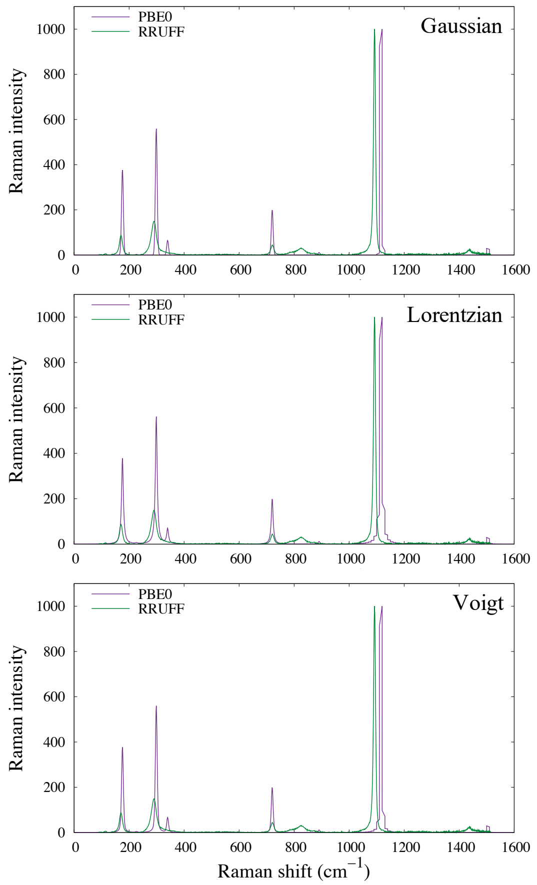

Supplementary Figure 1. Comparison of Raman spectra of $CaMg(CO_3)_2$ plotted using different line shapes. Raman spectra in all the other figures are plotted using a Voigt line shape with 50% Lorentzian and 50% Gaussian. On our web interface, plots of Raman spectra can be adjusted interactively using Gaussian, Lorentzian, and Voigt line shapes.

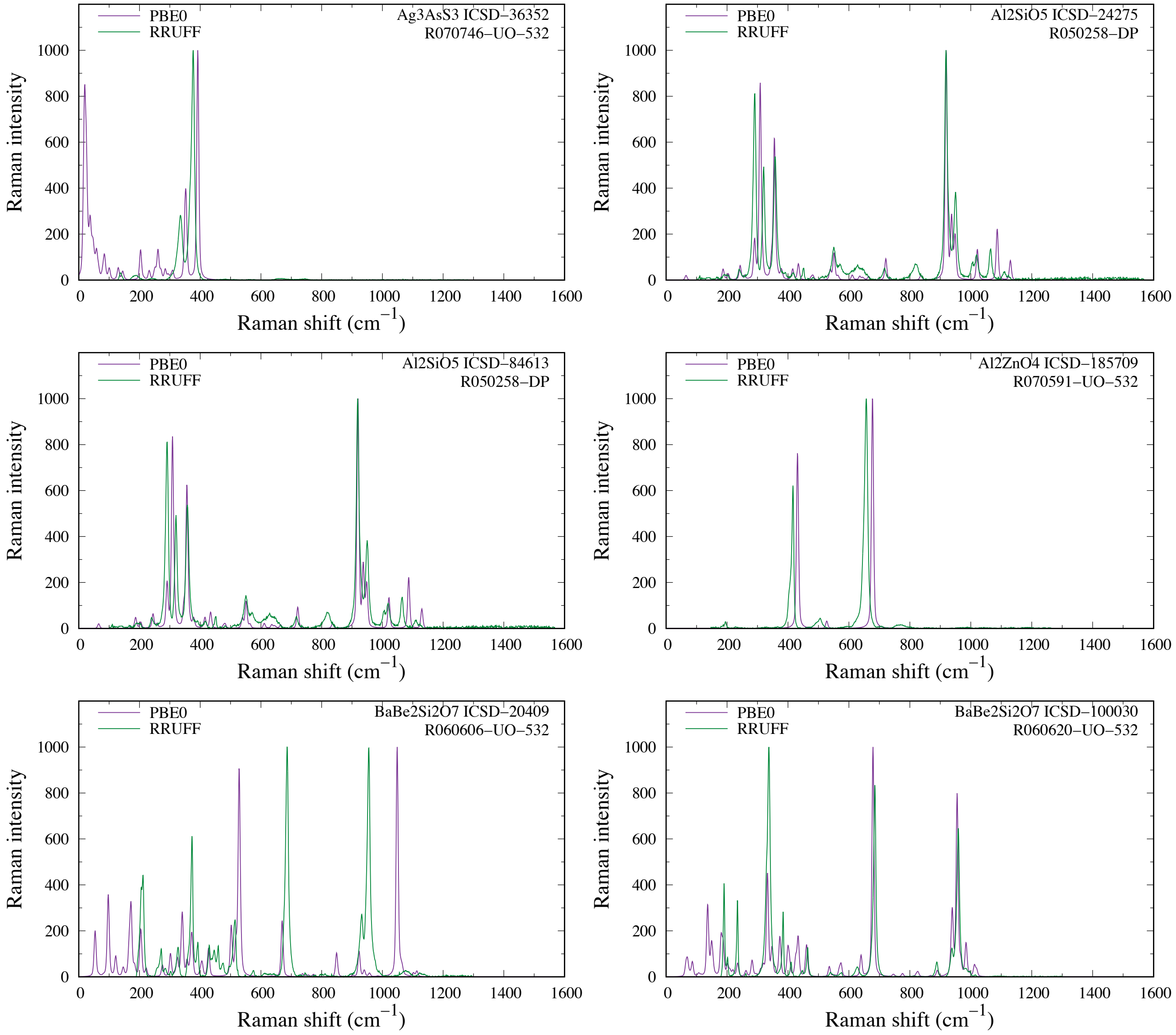

Supplementary Figure 2. Comparison of calculated (PBE0) and experimental (RRUFF) Raman spectra. The top right label shows chemical formula, ICSD number, and RRUFF ID; “UO” indicates unoriented sample, “DP” indicates depolarized incident laser and oriented sample, and the value after “UO” is the wavelength of incident laser in nanometer.

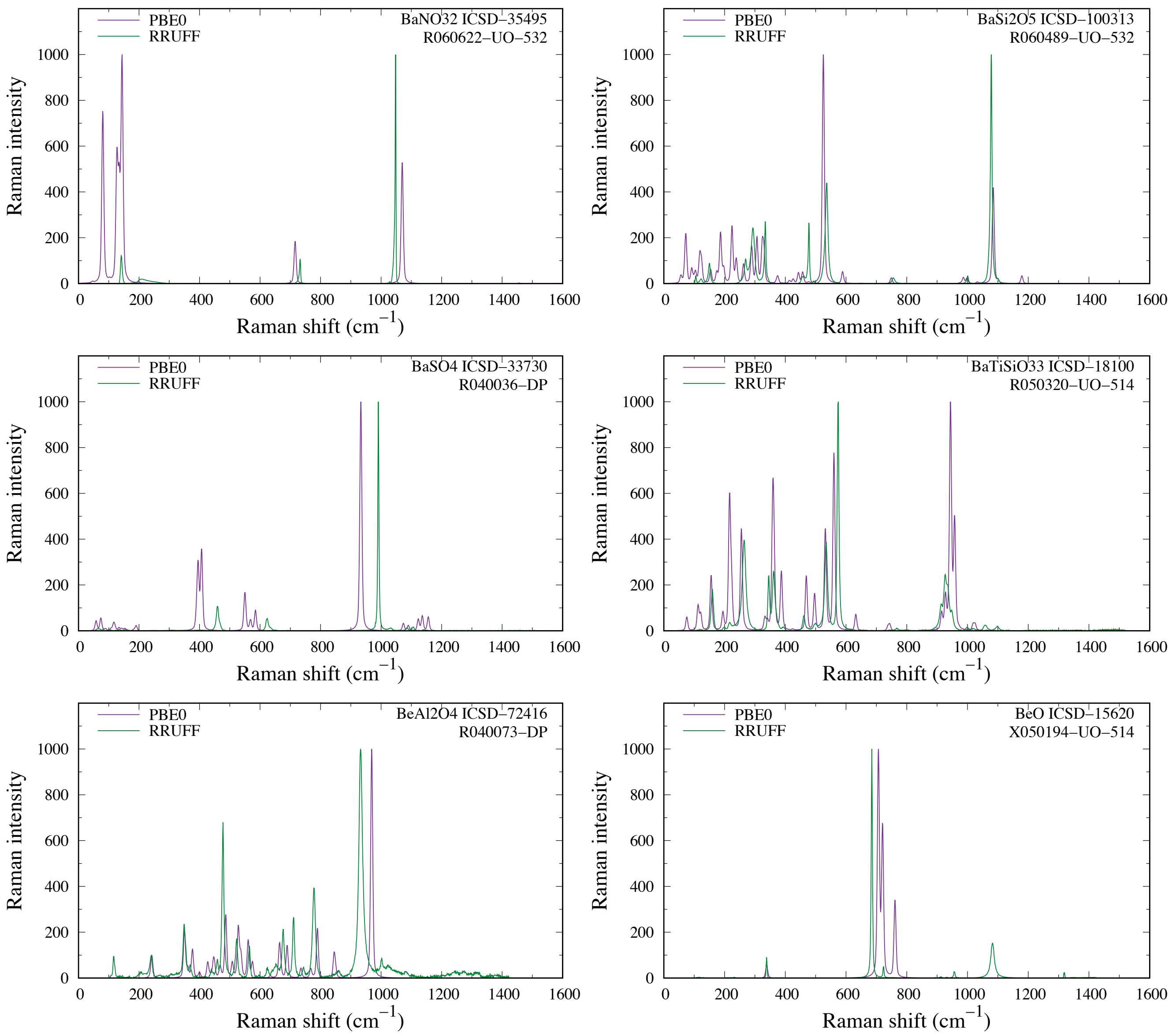

Supplementary Figure 3. Comparison of calculated (PBE0) and experimental (RRUFF) Raman spectra. The top right label shows chemical formula, ICSD number, and RRUFF ID; "UO" indicates unoriented sample, "DP" indicates depolarized incident laser and oriented sample, and the value after "UO" is the wavelength of incident laser in nanometer.

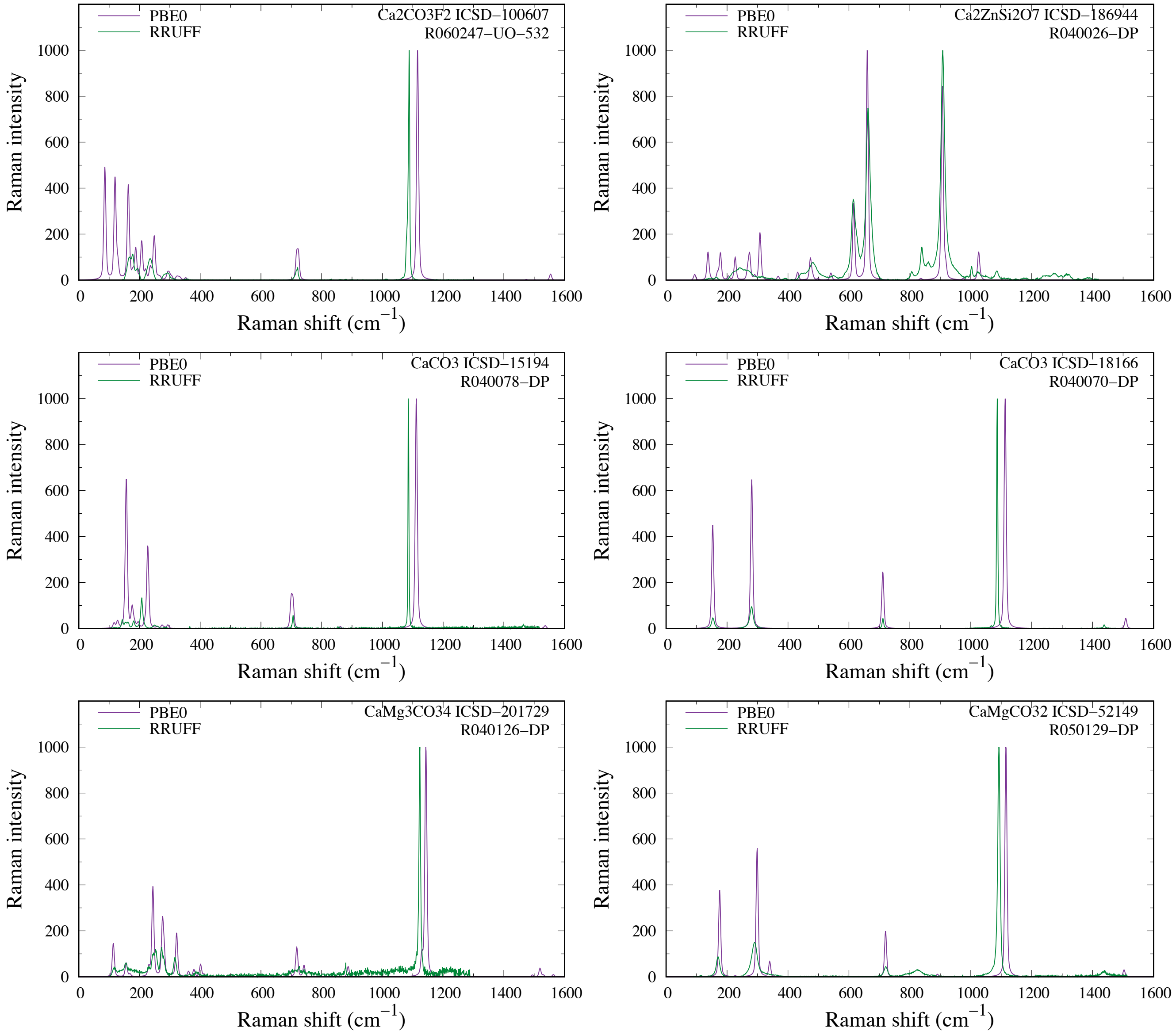

Supplementary Figure 4. Comparison of calculated (PBE0) and experimental (RRUFF) Raman spectra. The top right label shows chemical formula, ICSD number, and RRUFF ID; “UO” indicates unoriented sample, “DP” indicates depolarized incident laser and oriented sample, and the value after “UO” is the wavelength of incident laser in nanometer.

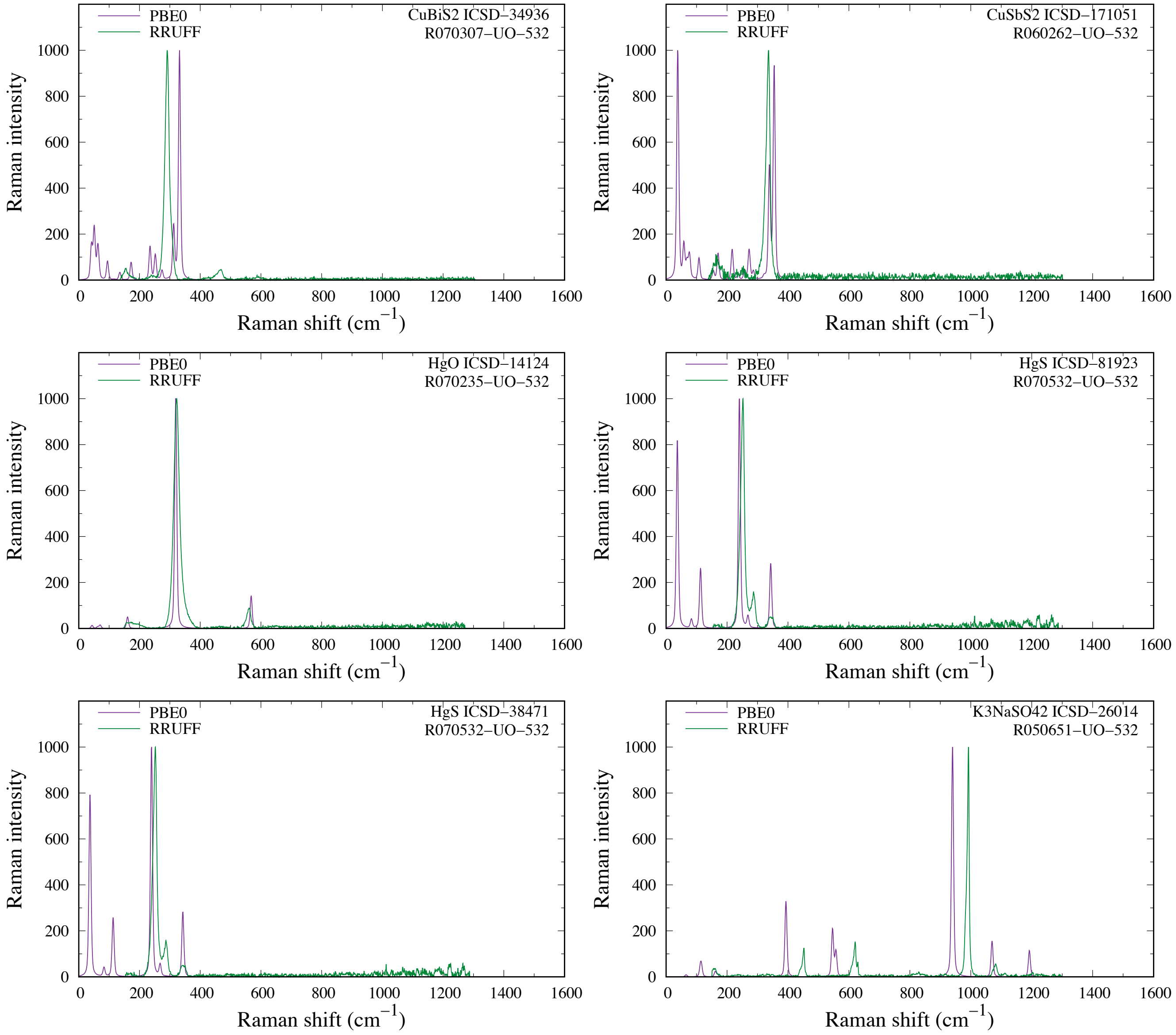

Supplementary Figure 5. Comparison of calculated (PBE0) and experimental (RRUFF) Raman spectra. The top right label shows chemical formula, ICSD number, and RRUFF ID; “UO” indicates unoriented sample, “DP” indicates depolarized incident laser and oriented sample, and the value after “UO” is the wavelength of incident laser in nanometer.

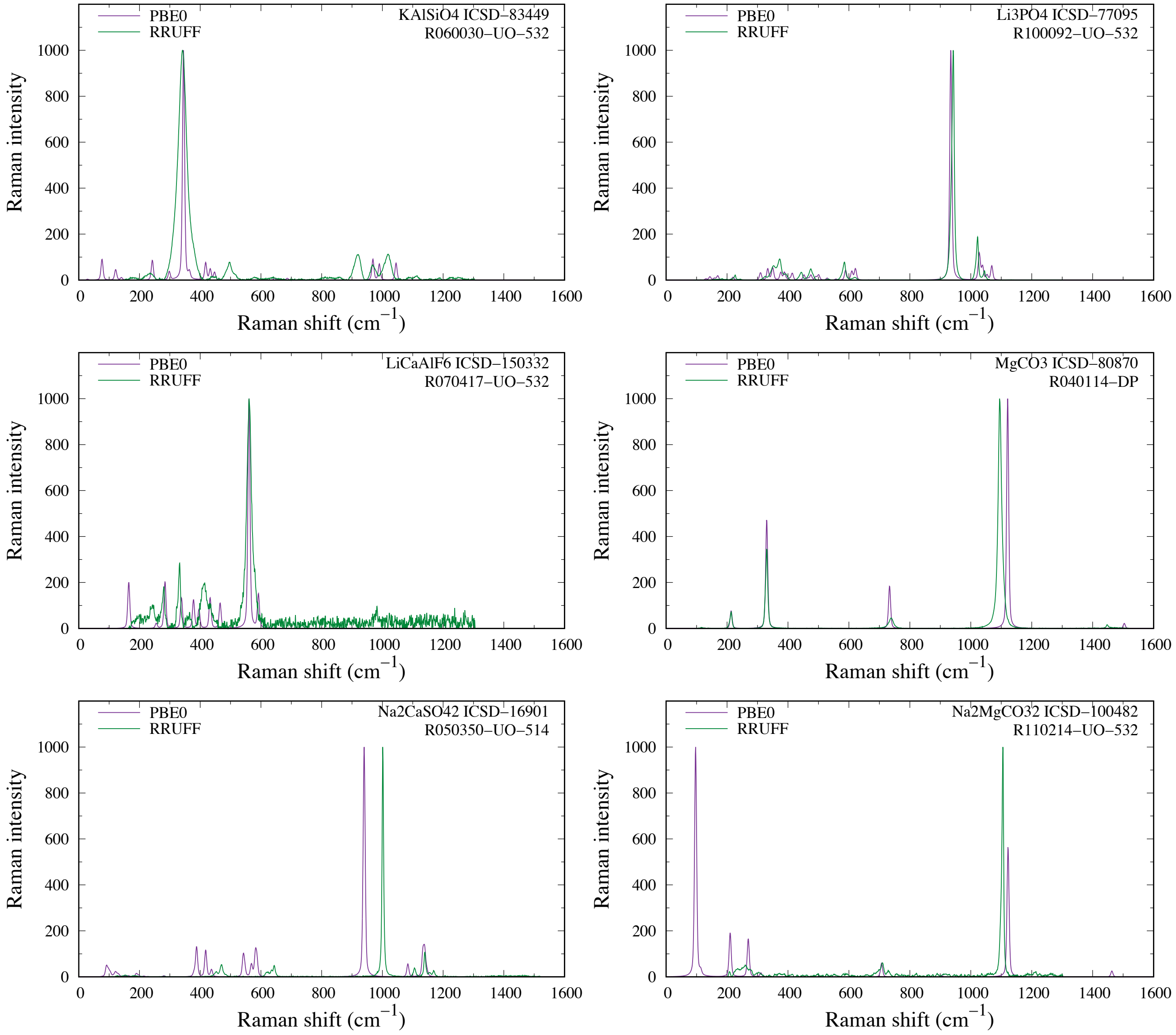

Supplementary Figure 6. Comparison of calculated (PBE0) and experimental (RRUFF) Raman spectra. The top right label shows chemical formula, ICSD number, and RRUFF ID; "UO" indicates unoriented sample, "DP" indicates depolarized incident laser and oriented sample, and the value after "UO" is the wavelength of incident laser in nanometer.

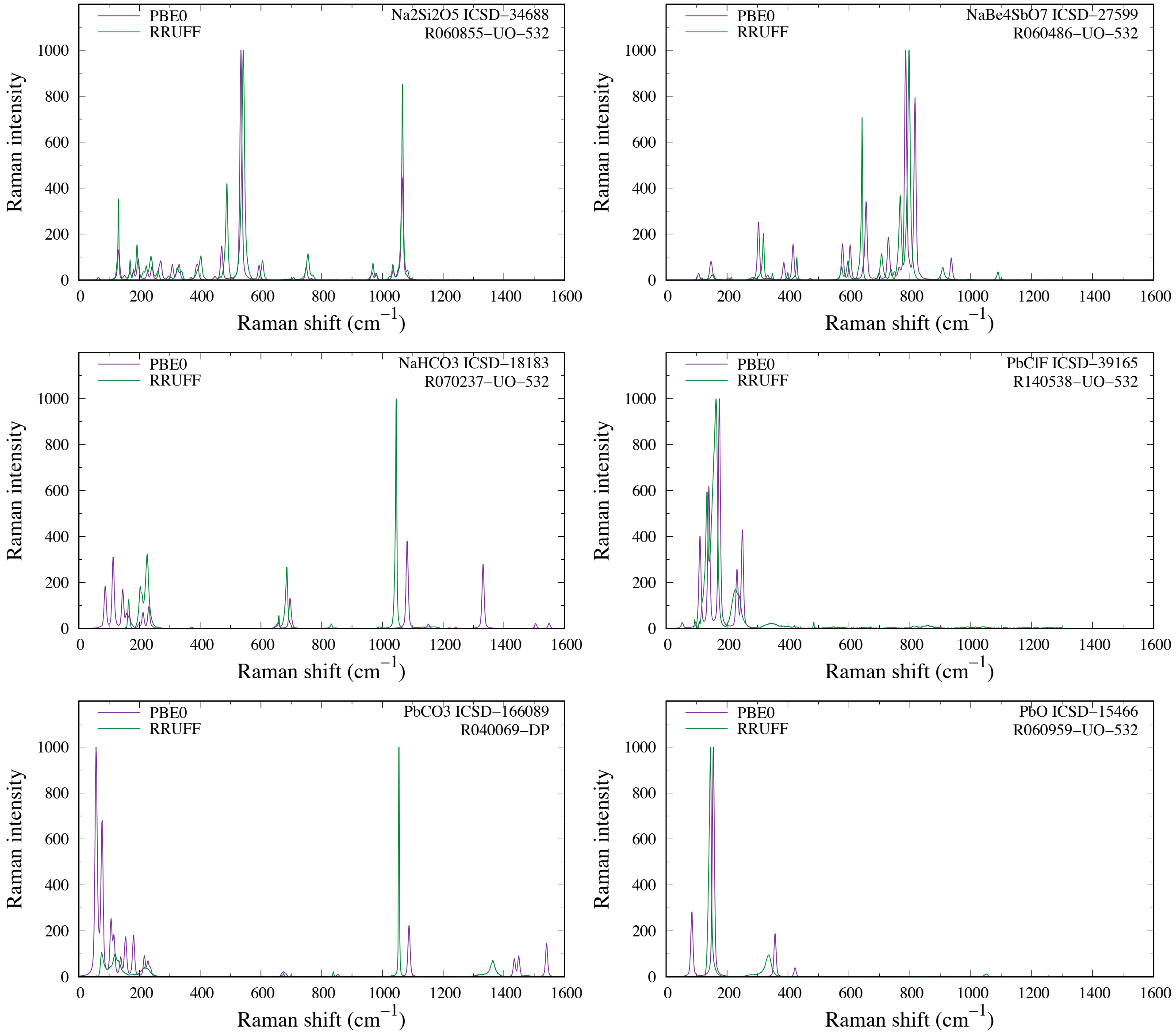

Supplementary Figure 7. Comparison of calculated (PBE0) and experimental (RRUFF) Raman spectra. The top right label shows chemical formula, ICSD number, and RRUFF ID; "UO" indicates unoriented sample, "DP" indicates depolarized incident laser and oriented sample, and the value after "UO" is the wavelength of incident laser in nanometer.

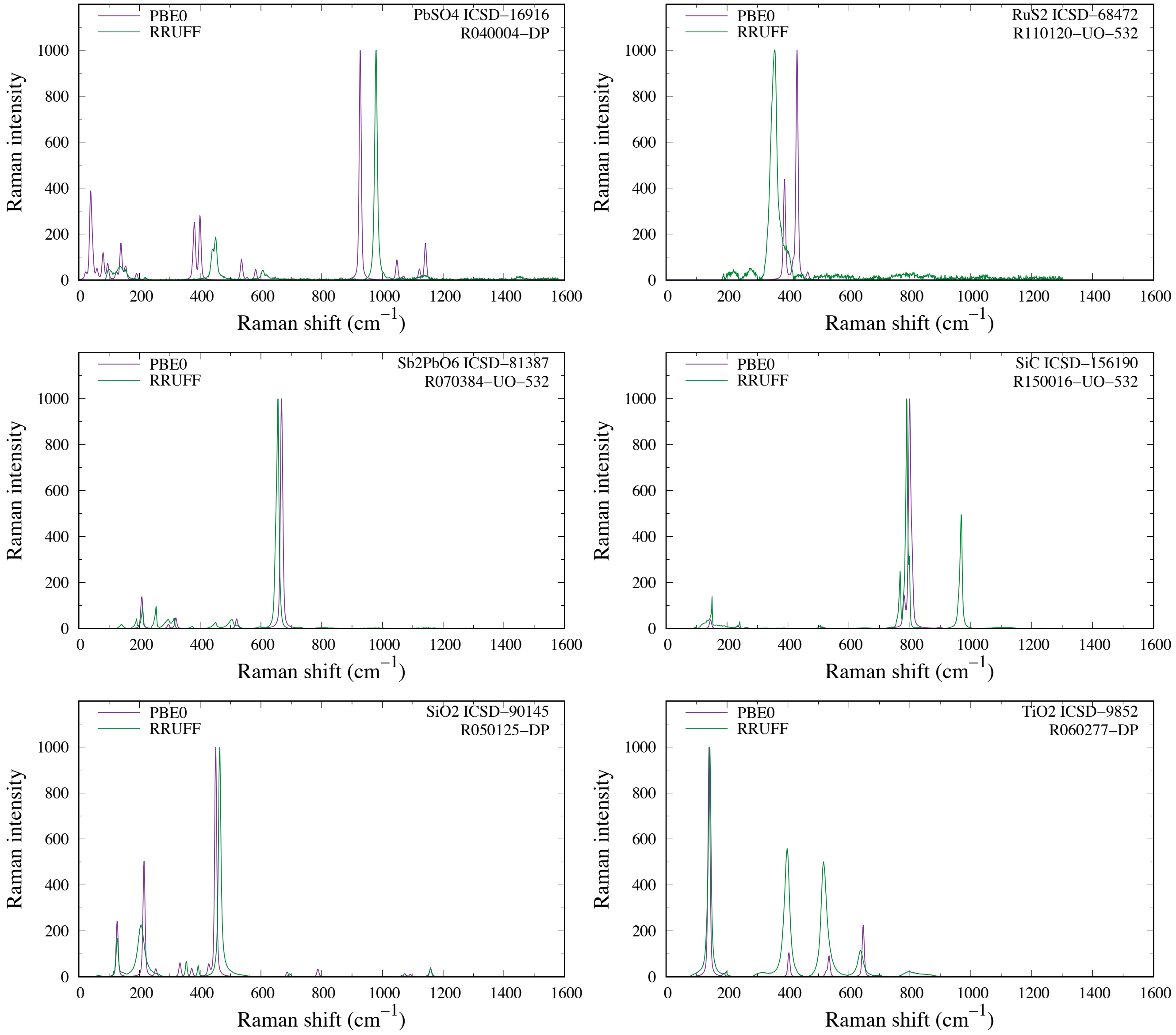

Supplementary Figure 8. Comparison of calculated (PBE0) and experimental (RRUFF) Raman spectra. The top right label shows chemical formula, ICSD number, and RRUFF ID; "UO" indicates unoriented sample, "DP" indicates depolarized incident laser and oriented sample, and the value after "UO" is the wavelength of incident laser in nanometer.

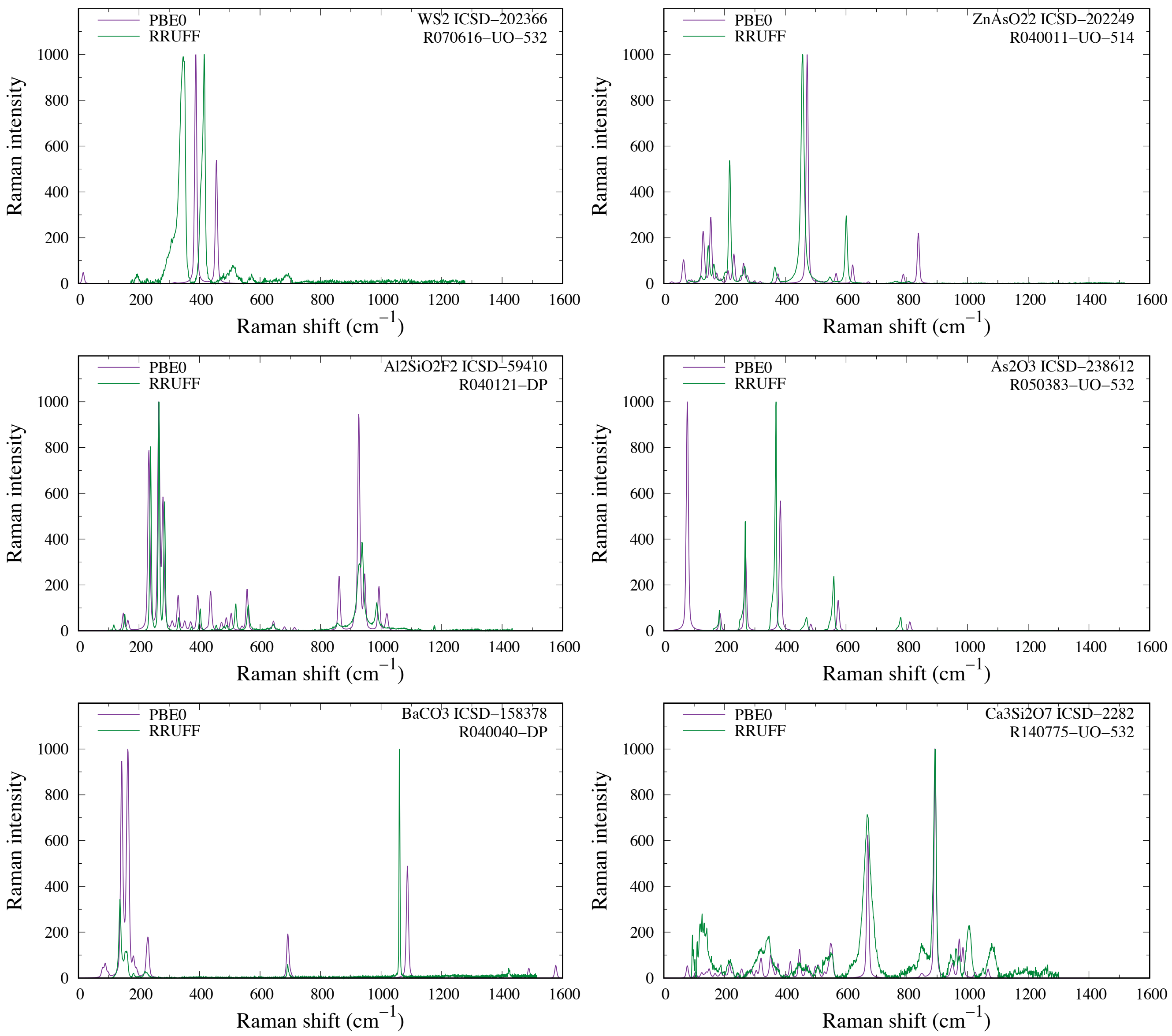

Supplementary Figure 9. Comparison of calculated (PBE0) and experimental (RRUFF) Raman spectra. The top right label shows chemical formula, ICSD number, and RRUFF ID; “UO” indicates unoriented sample, “DP” indicates depolarized incident laser and oriented sample, and the value after “UO” is the wavelength of incident laser in nanometer.

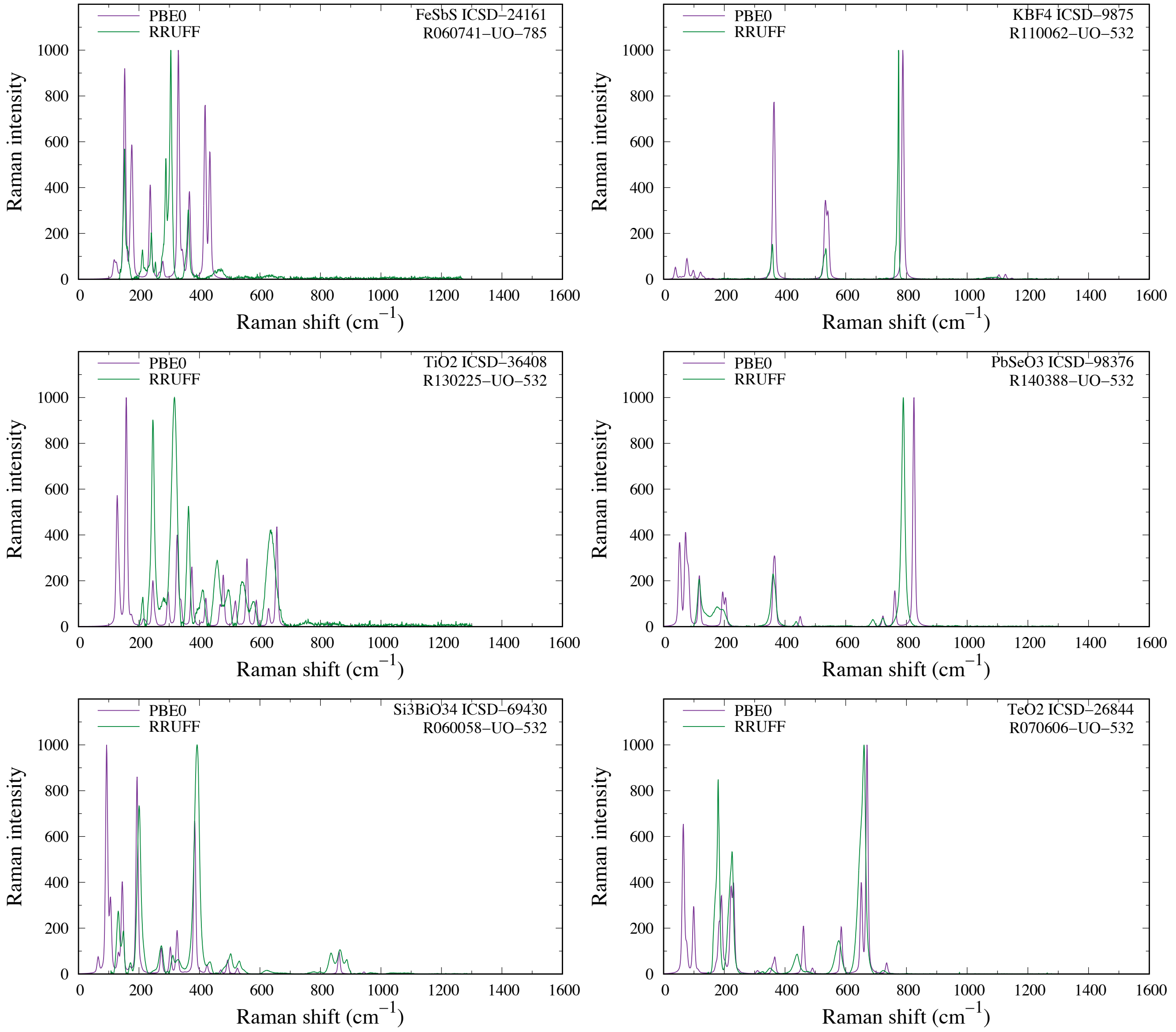

Supplementary Figure 10. Comparison of calculated (PBE0) and experimental (RRUFF) Raman spectra. The top right label shows chemical formula, ICSD number, and RRUFF ID; "UO" indicates unoriented sample, "DP" indicates depolarized incident laser and oriented sample, and the value after "UO" is the wavelength of incident laser in nanometer.

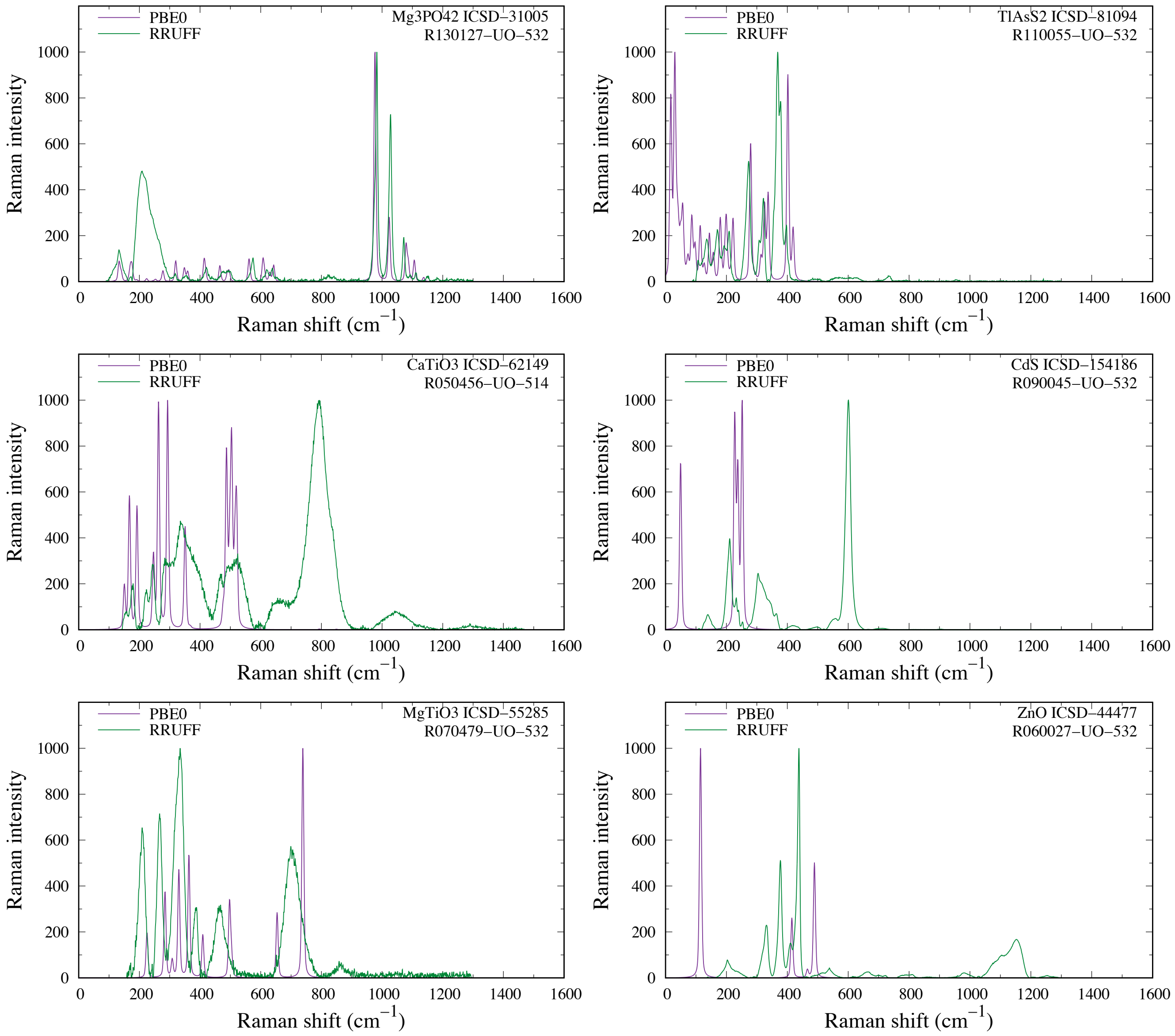

Supplementary Figure 11. Comparison of calculated (PBE0) and experimental (RRUFF) Raman spectra. The top right label shows chemical formula, ICSD number, and RRUFF ID; “UO” indicates unoriented sample, “DP” indicates depolarized incident laser and oriented sample, and the value after “UO” is the wavelength of incident laser in nanometer.

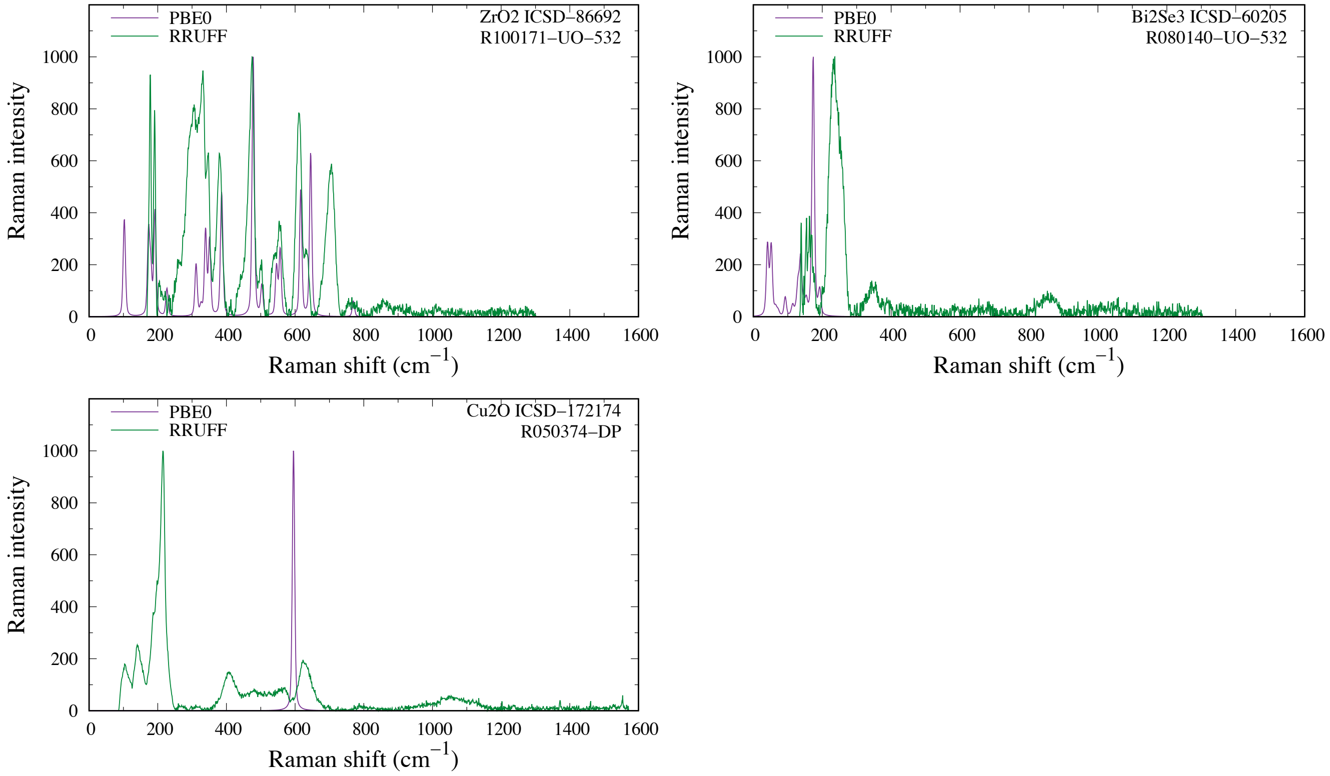

Supplementary Figure 12. Comparison of calculated (PBE0) and experimental (RRUFF) Raman spectra. The top right label shows chemical formula, ICSD number, and RRUFF ID; “UO” indicates unoriented sample, “DP” indicates depolarized incident laser and oriented sample, and the value after “UO” is the wavelength of incident laser in nanometer.

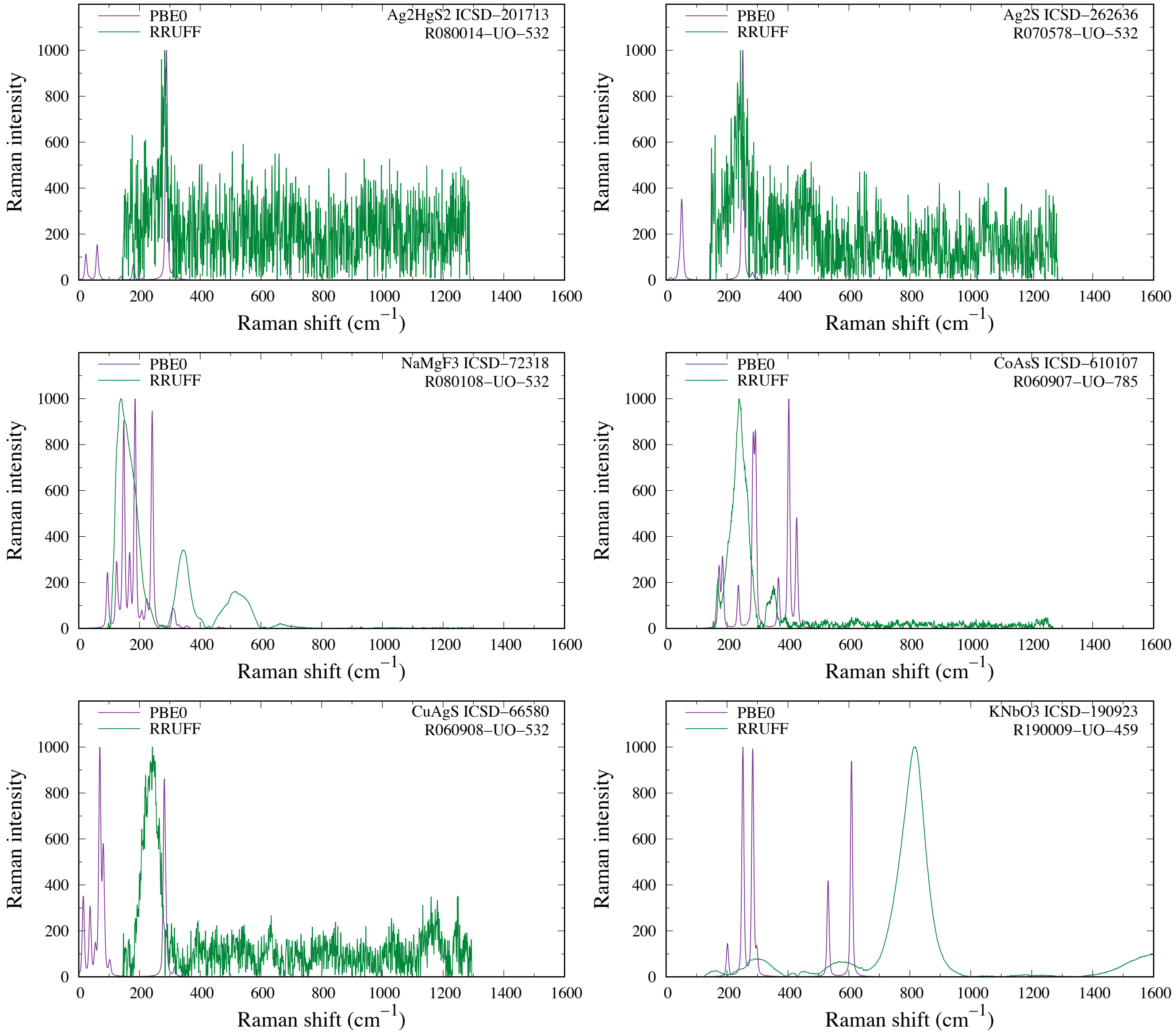

Supplementary Figure 13. Comparison of calculated (PBE0) and experimental (RRUFF) Raman spectra. These experimental spectra are either affected by device artifacts and are not meaningful to match with computation, or with peaks too much broadened and cannot be matched to computation.

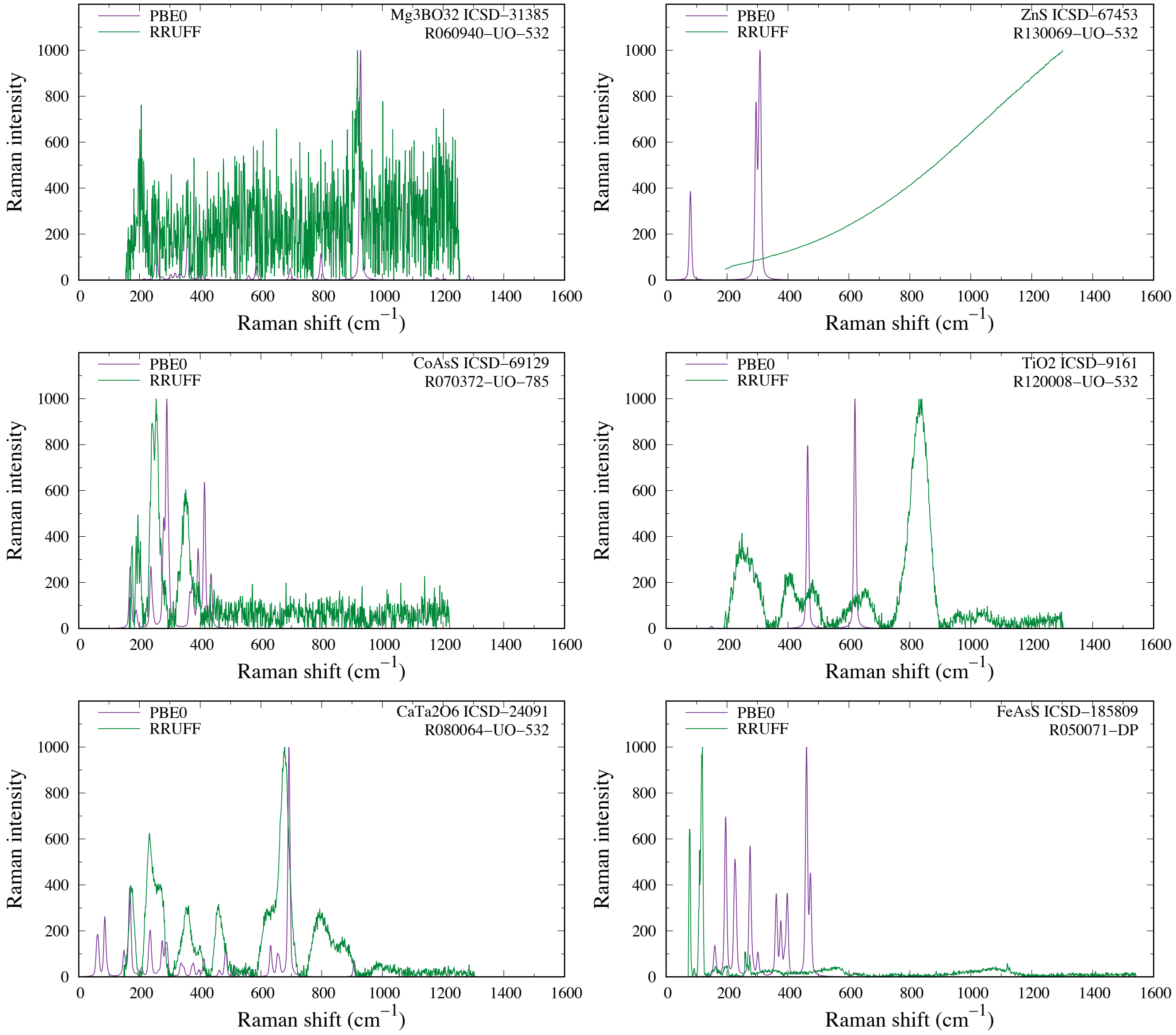

Supplementary Figure 14. Comparison of calculated (PBE0) and experimental (RRUFF) Raman spectra. These experimental spectra are either affected by device artifacts and are not meaningful to match with computation, or with peaks too much broadened and cannot be matched to computation.

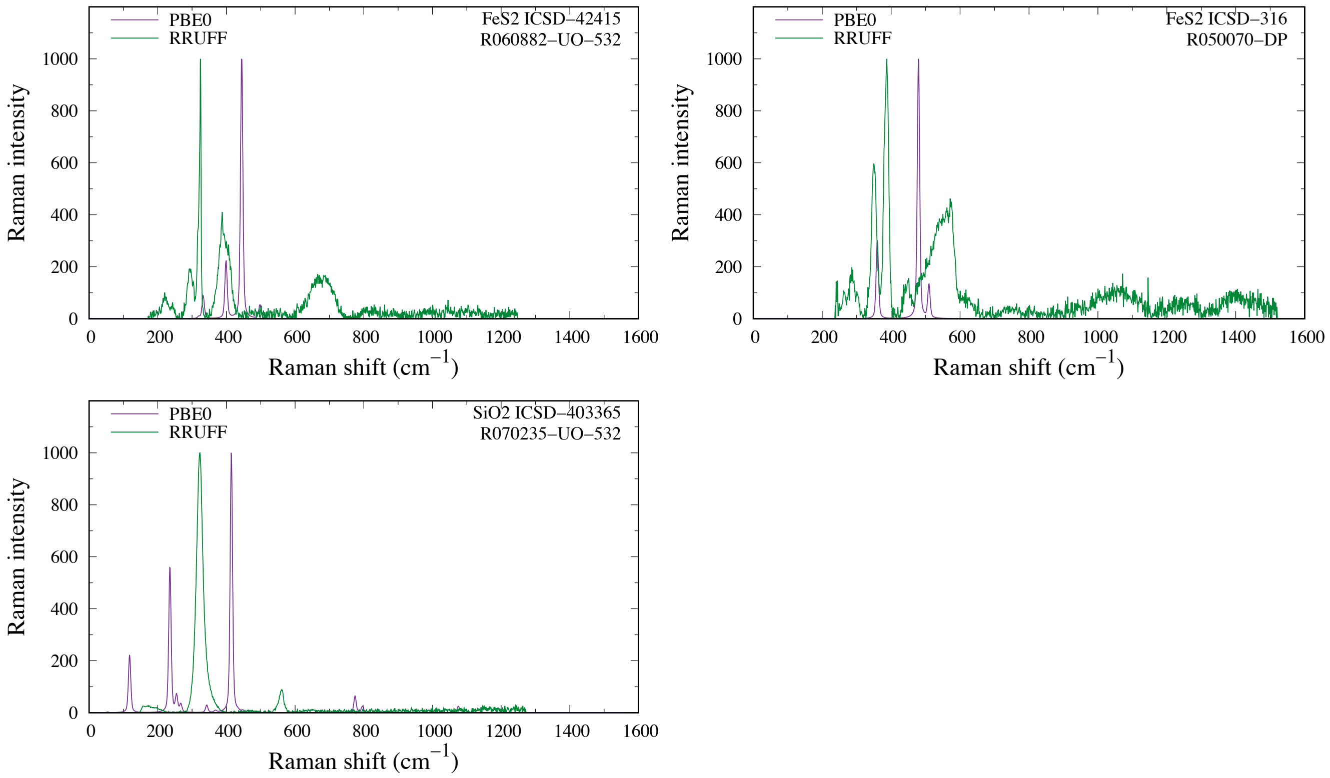

Supplementary Figure 15. Comparison of calculated (PBE0) and experimental (RRUFF) Raman spectra. These experimental spectra are either affected by device artifacts and are not meaningful to match with computation, or with peaks too much broadened and cannot be matched to computation.

Supplementary Tables

Ag_POB_TZVP_rev2	Al_pob_TZVP_rev2	As_pob_TZVP_rev2	B_pob_TZVP_rev2
Ba_pob_TZVP_rev2	Be_pob_TZVP_rev2	Bi_pob_TZVP_rev2	Br_pob_TZVP_rev2
C_pob_TZVP_rev2	Ca_pob_TZVP_rev2	Cd_POB_TZVP_rev2	Cl_pob_TZVP_rev2
Co_pob_TZVP_rev2	Cu_pob_TZVP_rev2	F_pob_TZVP_rev2	Fe_pob_TZVP_rev2
Ge_pob_TZVP_rev2	H_pob_TZVP_rev2	Hg_pob_TZVP_rev2	I_POB_TZVP_2018
K_pob_TZVP_rev2	Li_pob_TZVP_rev2	Mg_pob_TZVP_rev2	N_pob_TZVP_rev2
Na_pob_TZVP_rev2	Nb_POB_TZVP_rev2	O_pob_TZVP_rev2	P_pob_TZVP_rev2
Pb_pob_TZVP_rev2	Ru_POB_TZVP_rev2	S_pob_TZVP_rev2	Sb_POB_TZVP_2018
Se_pob_TZVP_rev2	Si_pob_TZVP_rev2	Sn_POB_TZVP_rev2	Ta_pob_TZVP_rev2
Te_POB_TZVP_2018	Ti_pob_TZVP_rev2	Tl_pob_TZVP_rev2	W_pob_TZVP_rev2
Zn_pob_TZVP_rev2	Zr_POB_TZVP_2018		

Supplementary Table 1. List of the TZVP basis sets for elements in the computed inorganic compounds. For some elements, the pob-TZVP-rev2 basis sets are not available and an older version of the TZVP basis sets were used. The basis-set files can be found on the CRYSTAL website (https://www.crystal.unito.it/basis_sets.html) with exactly the same name as in this list.
